# A putative bHLH transcription factor is a candidate gene for *male sterile 32*, a locus affecting pollen and tapetum development in tomato

**DOI:** 10.1038/s41438-019-0170-2

**Published:** 2019-07-21

**Authors:** Xiaoyan Liu, Mengxia Yang, Xiaolin Liu, Kai Wei, Xue Cao, Xiaotian Wang, Xiaoxuan Wang, Yanmei Guo, Yongchen Du, Junming Li, Lei Liu, Jinshuai Shu, Yong Qin, Zejun Huang

**Affiliations:** 1 College of Forestry and Horticulture, Xinjiang Agricultural University, 830052 Urumqi, China; 2 Key Laboratory of Biology and Genetic Improvement of Horticultural Crops of the Ministry of Agriculture, Institute of Vegetables and Flowers, Chinese Academy of Agricultural Sciences, 100086 Beijing, China

**Keywords:** Pollen, Plant breeding

## Abstract

The tomato (*Solanum lycopersicum*) *male sterile 32* (*ms32*) mutant has been used in hybrid seed breeding programs largely because it produces no pollen and has exserted stigmas. In this study, histological examination of anthers revealed dysfunctional pollen and tapetum development in the *ms32* mutant. The *ms32* locus was fine mapped to a 28.5 kb interval that encoded four putative genes. *Solyc01g081100*, a homolog of Arabidopsis *bHLH10/89/90* and rice *EAT1*, was proposed to be the candidate gene of *MS32* because it contained a single nucleotide polymorphism (SNP) that led to the formation of a premature stop codon. A codominant derived cleaved amplified polymorphic sequence (dCAPS) marker, MS32D, was developed based on the SNP. Real-time quantitative reverse-transcription PCR showed that most of the genes, which were proposed to be involved in pollen and tapetum development in tomato, were downregulated in the *ms32* mutant. These findings may aid in marker-assisted selection of *ms32* in hybrid breeding programs and facilitate studies on the regulatory mechanisms of pollen and tapetum development in tomato.

## Introduction

Tomato (*Solanum lycopersicum*) is one of the most important vegetable crops in the world. Hybrid vigor has been extensively utilized in tomato production because F_1_-hybrid cultivars usually exhibit a greater yield and higher disease resistance than open-pollinated varieties^1,2^. Male sterility has proven to be a useful trait for hybrid seed production^3^. Male-sterile mutants are classified as structural, functional, and sporogenous types^4^. Structural male-sterile mutants of tomato usually bear extremely deformed stamens and do not produce pollen, such as *stamenless* (*sl*)^5^ and *sl-2*^6^. Functional male-sterile mutants can produce normal pollen, but the pollen cannot reach the stigma because of indehiscent anthers or dehiscent anthers with morphological abnormalities, such as *positional sterile* (*ps*)^7^, *ps-2*^8^, and *exserted* stigma (*ex*)^9^. Sporogenous male-sterile mutants exhibit relatively normal floral morphology but produce little or no fertile pollen. Most tomato male-sterile mutants belong to this type^10^. Thus, fine mapping and identifying the genes for sporogenous male-sterile mutants of tomato will not only help us understand the regulation of pollen development but also facilitate the use of these mutants in hybrid seed breeding programs.

Pollen development has been widely studied in Arabidopsis and rice (*Oryza sativa*)^11^. Several transcription factor genes have been identified as regulators involved in pollen development in Arabidopsis, such as *DYSFUNCTIONAL TAPETUM1* (*DYT1*, referred to as *AtDYT1* hereafter), *DEFECTIVE TAPETUM DEVELOPMENT AND FUNCTION1* (*TDF1*, *AtTDF1*, syn. *AtMYB35*), *ABORTED MICROSPORES* (*AMS*, *AtAMS*), basic helix-loop-helix 10 (*bHLH10*, *AtbHLH10*), *bHLH89* (*AtbHLH89*), *bHLH91* (*AtbHLH91*), and *MYB80* (*AtMYB80*, syn. *AtMYB103*)^12–17^. *AtDYT1* encodes a bHLH transcription factor that directly regulates the expression of *AtTDF1*^18^. *AtTDF1* affects the expression of *AtAMS*^19^. AtAMS protein can interact with AtbHLH89/91 and regulate the expression of *AtMYB80*^20,21^. The interaction between AtDYT1 and AtbHLH10/89/91 is also important for the regulation of pollen development^22^. Therefore, they form a *DYT1*-*TDF1*-*AMS*-*bHLH10/89/91*-*MYB80* transcriptional cascade to precisely regulate pollen and tapetum development in Arabidopsis^17,23,24^. A similar signaling pathway including the *UDT1*-*TDR1*-*TIP2*-*EAT1* transcriptional cascade is also involved in the regulation of pollen and tapetum development in rice^18^. *UDT1* (*UNDEVELOPED TAPETUM1*, *OsUDT1*) is a homolog of *AtDYT1*^25^, *TDR1* (*TAPETUM DEGENERATION RETARDATION1*, *OsTDR1*) is a homolog of *AtAMS*^26^, and *TIP2* (*TDR INTERACTING PROTEIN2*, *OsTIP2*) and *EAT1* (*ETERNAL TAPETUM 1*, *OsEAT1*) are homologs of *AtbHLH10/89/90*^18,27^. These findings suggest that the signaling pathway regulating pollen and tapetum development is highly conserved in higher plants^23^. In tomato, until now, only one regulator gene, *SlMS10*, a homolog of *AtDYT1* and *OsUDT1*, has been found to be involved in pollen and tapetum development. A putative regulatory network for pollen and tapetum development in tomato, which is similar to the networks in Arabidopsis and rice, was presented based on the evolutionary relationships and conserved functions of the genes differentially expressed between the wild type (WT) and *male sterile 10*^*35*^ (*ms10*^*35*^)^24^. However, except for *SlMS10*, the regulators in the network need further investigation.

The tomato *ms32* mutant is completely sterile because it does not produce pollen^28^. It has been used in hybrid seed breeding programs^3^. In this study, histological examination revealed dysfunctional microgametogenesis and tapetum degradation in the *ms32* mutant. The *ms32* locus was fine mapped to a 28.5 kb interval that encoded four putative genes. *Solyc01g081100* was proposed to be the candidate gene of *MS32* as it contained a single nucleotide polymorphism (SNP) that resulted in the formation of a premature stop codon. *Solyc01g081100* is a homolog of *AtbHLH10/89/90* and *OsEAT1*. Real-time quantitative reverse-transcription PCR (qPCR) showed that most of the regulator genes, which were proposed to be involved in pollen and tapetum development, were downregulated in the *ms32* mutant. However, the expression of *SlMS10* did not show a significant difference in the *ms32* mutant compared to the WT. These findings may help in marker-assisted selection of *ms32* in hybrid breeding programs and facilitate studies on the regulatory mechanisms of pollen and tapetum development in tomato.

## Results

### Phenotypic investigation of the *ms32* mutant

**Fig. 1:**
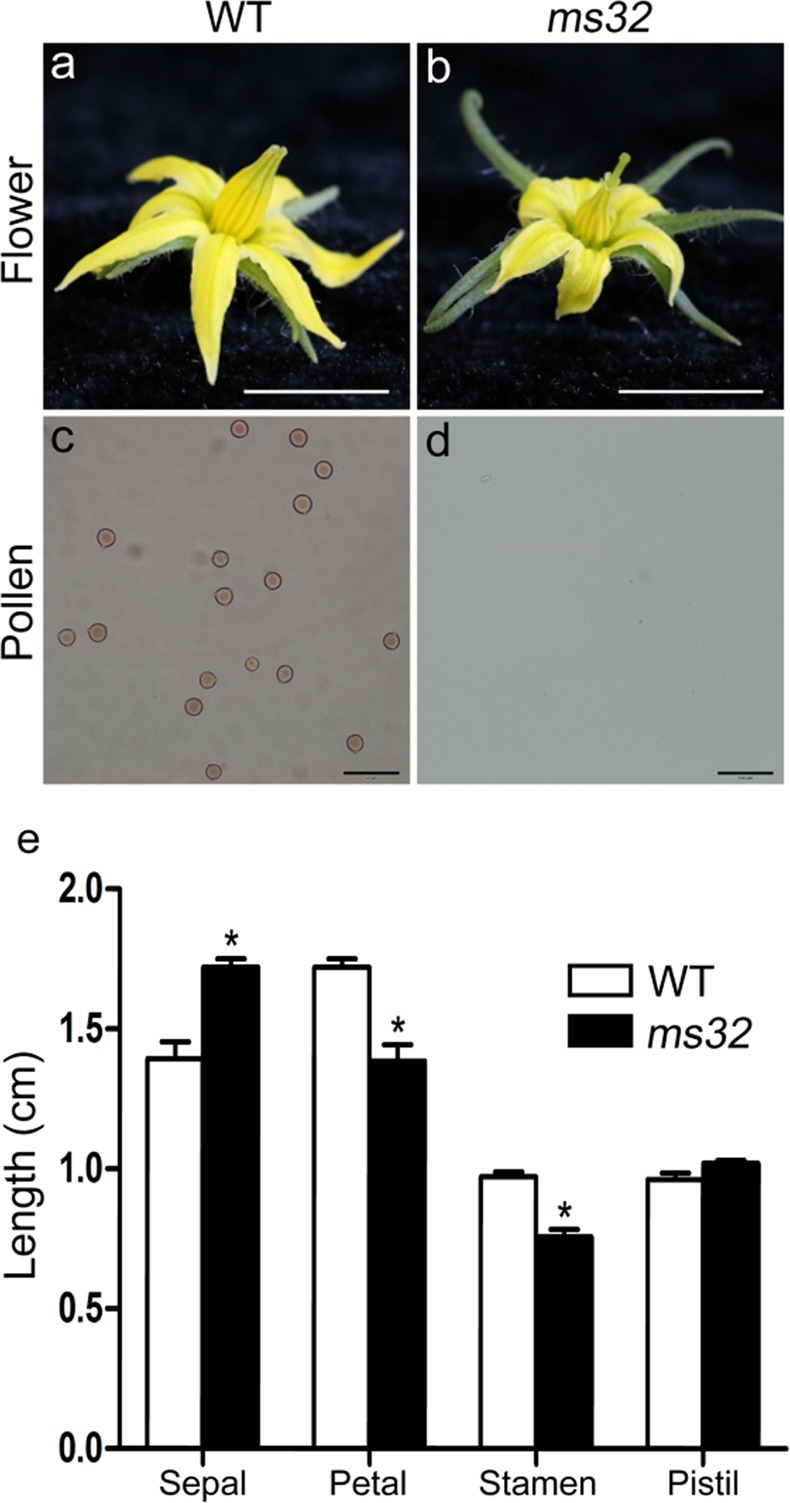
Flower phenotypes and pollen production of the *ms32* mutant. **a**, **b** Flower of WT (**a**) and *ms32* plants (**b**). Scale bars, 1 cm. **c**, **d** Analysis of pollen viability by acetocarmine staining of pollen from anthers of WT (**c**) and *ms32* plants (**d**). Scale bars, 50 μm. **e** Length of floral organs in WT and *ms32* flowers at anthesis. Values represent the means ± SEs. Asterisks indicate a significant difference (*p* < 0.05) between the WT and *ms32* mutant

The morphologies of the *ms32* mutant and WT plants were the same until the flowering stage. At the flowering stage, the *ms32* mutant had longer sepals and shorter petals than the WT (Fig. 1). In addition, the stamens in *ms32* flowers were greatly reduced and shrunken and pale in color, usually with the stigma remaining exserted (Fig. 1). Pollen viability analysis detected no pollen in the flowers of the *ms32* mutant (Fig. 1). The *ms32* mutant could not set fruit after self-pollination with an electric vibrator, but it was able to produce fruit when manually pollinated with pollen from WT plants (Fig. S1). These findings were consistent with a previous study of *ms32*^28^.

### Histological examination of *ms32* anthers

**Fig. 2:**
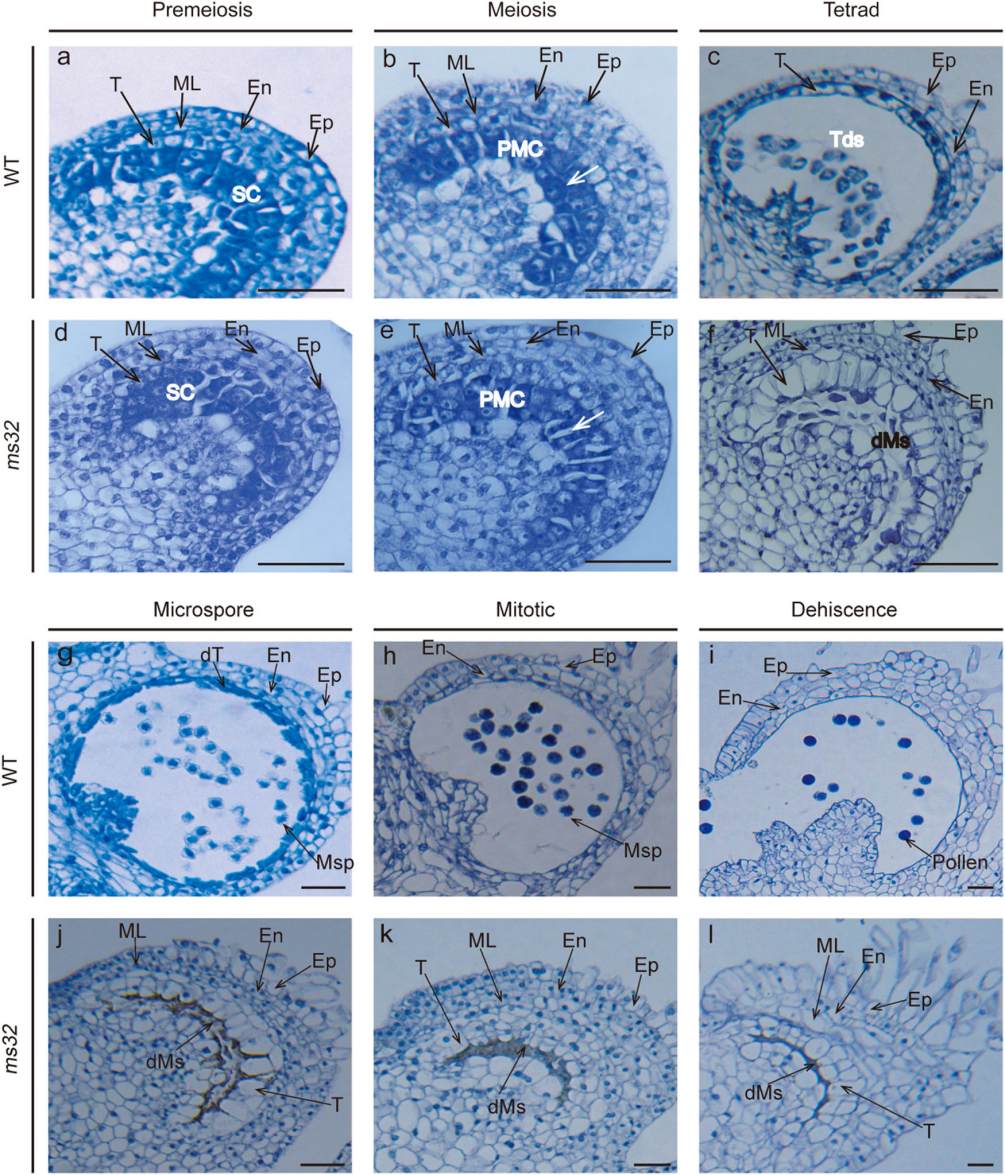
Histological examination of anthers at different developmental stages in WT and *ms32* plants. Transverse sections of WT (**a–c**, **g–i**) and *ms32* (**d–f**, **j–l**) anthers at different developmental stages. **a**, **d** Premeiotic stage; **b**, **e** Meiotic stage, with white arrows pointing to the crushed and separated PMCs; **c**, **f** Tetrad stage; **g**, **j** Microspore stage; **h**, **k** Mitotic stage; **i**, **l** Dehiscence stage. dMs, degenerated meiocytes; dT, degenerated tapetum; En, endothecium; Ep, epidermis; ML, middle cell layer; Msp, microspore; PMC, pollen mother cell; SC, sporogenous cell; T, tapetum; Tds, tetrads. Scale bars, 50 μm

Histological examination of anthers at different developmental stages was performed to detect the spatial and temporal occurrence of defects in *ms32* anthers. At the premeiotic stage, the cell layer differentiation in *ms32* anthers seemed to be similar to that in WT anthers (Fig. 2a, d). At the meiotic stage of WT anthers, sporogenous cells developed in pollen mother cells (PMCs), and meiosis was initiated and completed (Fig. 2b). However, PMCs began to crush and separate from each other in *ms32* anthers (Fig. 2e). At the tetrad, microspore, mitotic and dehiscence stages, dramatic changes in morphology were detected between WT and *ms32* anthers. In WT anthers, PMCs successively developed into tetrads (Fig. 2c), microspores (Fig. 2g), vacuolated microspores (Fig. 2h), and pollen grains (Fig. 2i). In addition, the tapetal cells gradually degenerated and disappeared (Fig. 2c, g–i). In *ms32* anthers, PMCs were crushed and could not produce tetrads, microspores and pollen grains successively. In contrast, tapetal cells were enlarged and vacuolated at the tetrad stage and remained until the dehiscence stage (Fig. 2f, j–l).

### Genetic analysis of the *ms32* locus

To determine the inheritance of the *ms32* locus, F_1_ plants were obtained from a cross between the *ms32* mutant and LA1589. All F_1_ plants developed normal flowers and fruits. The F_2_ population derived from the abovementioned F_1_ plants segregated in an approximate 3:1 ratio (fertility:sterility = 500:165, χ^2^ = 0.27, less than χ^2^_0.05_ = 3.84, χ^2^-test), which indicated that the sterility phenotype was caused by a single recessive mutation.

### Fine mapping of the *ms32* locus

**Fig. 3:**
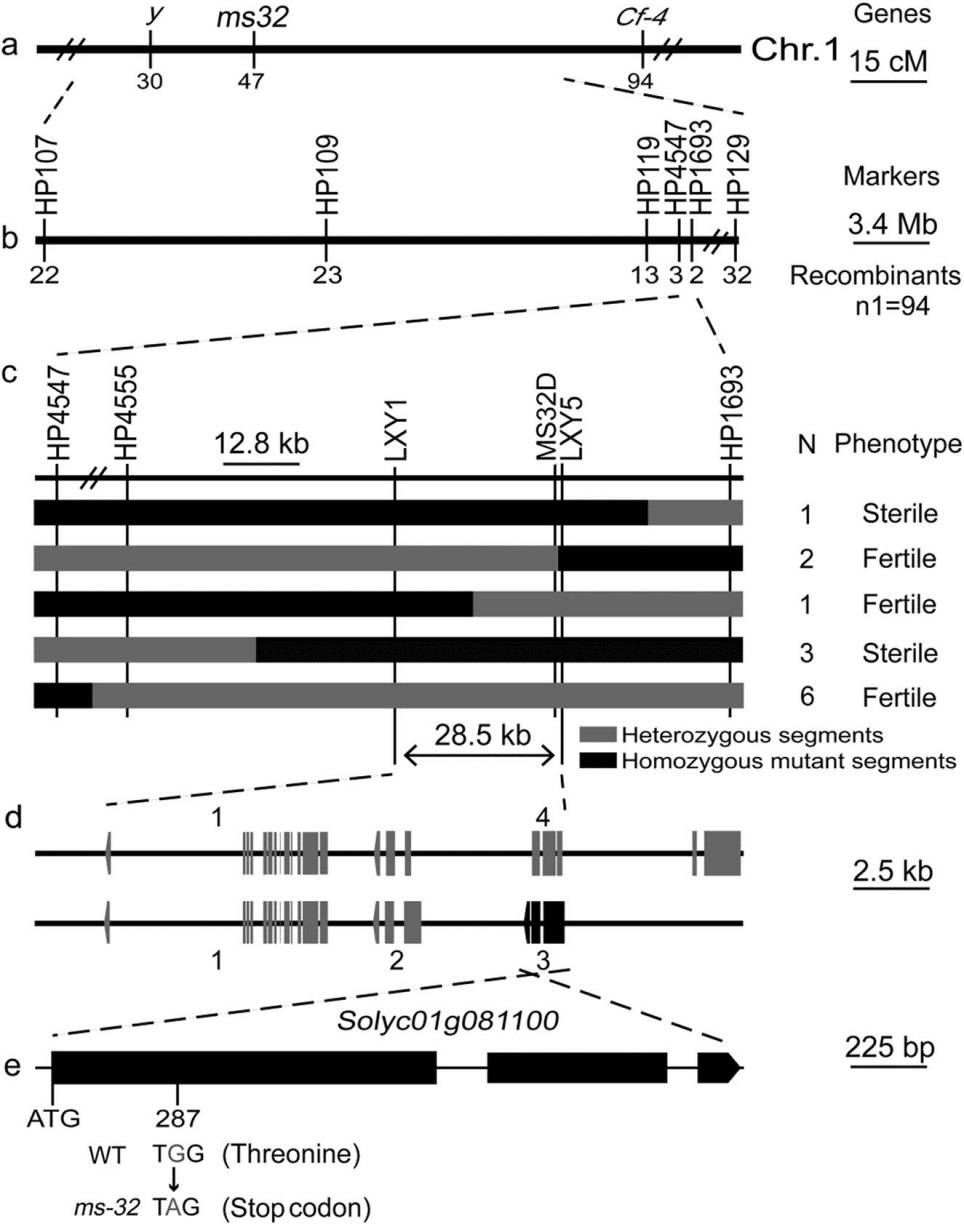
Fine mapping of the *ms32* locus. **a** Genetic position of the *ms32* locus on the genetic map constructed by Mutschler et al.^29^. **b** Preliminary mapping of the *ms32* locus using 94 sterile male plants. **c** Fine mapping of the *ms32* locus. N indicates the number of recombinants. **d** The annotated genes near the *ms32* locus according to ITAG release 3.2. Rectangles and arrows indicate the exons and direction of transcription, respectively. **e** The exon-intron structure of the coding region of *Solyc01g081100* and the sequence polymorphism in the *ms32* mutant

The *ms32* locus has been determined to be located between the *y* locus and *Cf-4* locus on chromosome 1 (Fig. 3a)^29^. The genes *Y* and *CF-4* have been cloned^30,31^. Based on this information, six insertion/deletion (InDel) markers were developed in the region between the two genes (Table S1). For preliminary mapping of the *ms32* locus, these InDel markers were used to genotype 94 sterile male plants in an F_2_ population. The genotype of the recombinant individuals, which was detected by these markers, indicated that the *ms32* locus was located between markers HP4547 and HP1693 (Fig. 3b). For fine mapping of the *ms32* locus, three InDel markers were developed within the region between markers HP4547 and HP1693. The markers HP4547 and HP1693 were utilized to genotype 665 individuals from the whole F_2_ population. A total of 13 recombinants, which were determined to be homozygous for the *ms32* mutant and for just one of these two markers, were selected for further genotyping. Based on the genotype and phenotype of these recombinants, the *ms32* locus was narrowed to an interval between markers LXY1 and LXY5, a 28.5 kb interval on chromosome 1 (Fig. 3c).

### Candidate gene analysis

**Fig. 4:**
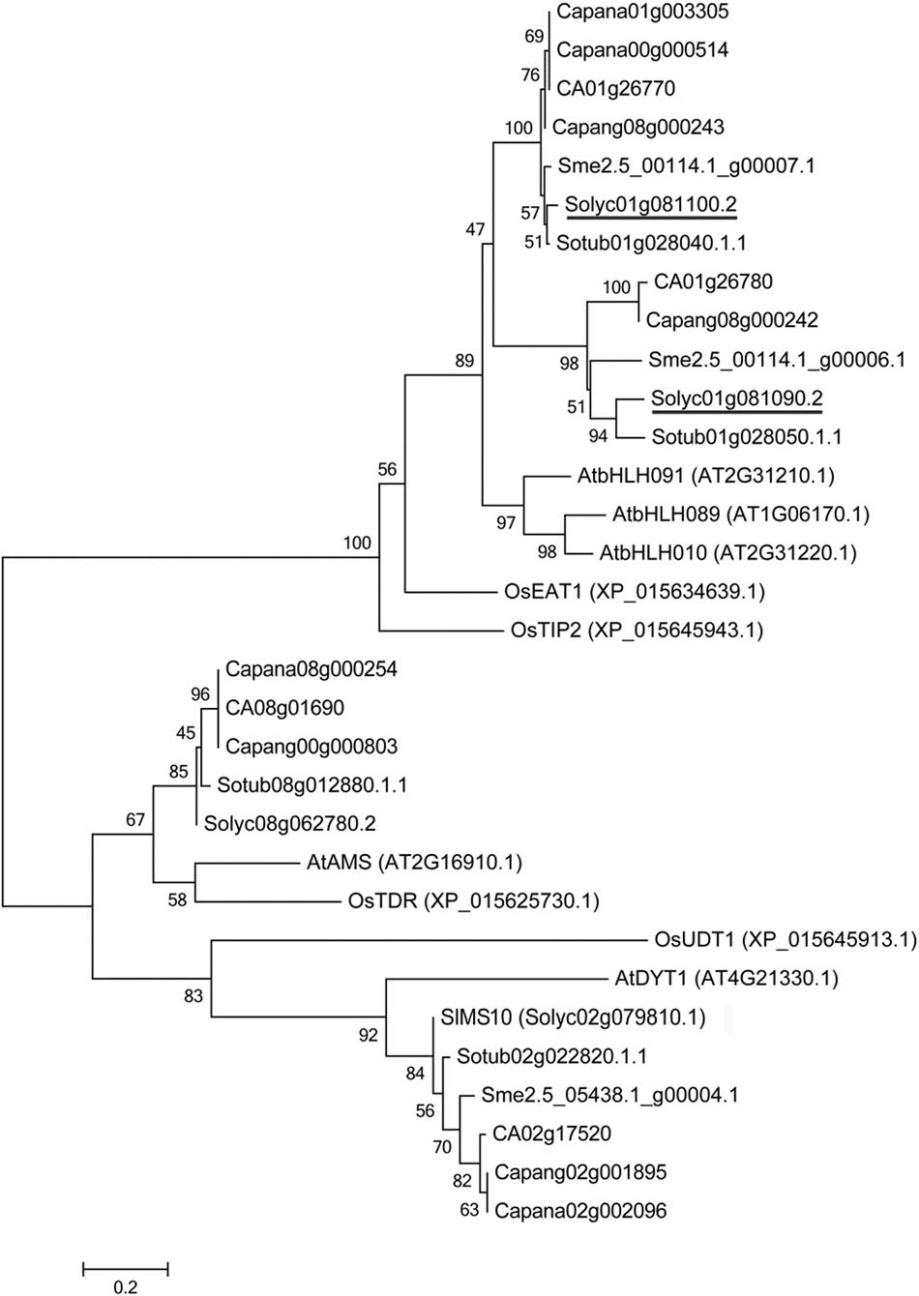
Phylogenetic analysis of bHLH proteins related to pollen and tapetum development in several plant species. The phylogenetic tree was constructed with the neighbor-joining method by using MEGA6 (Tamura et al., 2013) with 1000 bootstrap replicates, which was based on the full-length deduced amino acid sequences. Solyc01g081090.2 and Solyc01g081100.2 are underlined. At, *Arabidopsis thaliana*; Ca, *Capsicum annuum*; Capana, *Capsicum annuum* var. zunla; Capang, *Capsicum annuum* var. glabriusculum; Os, *Oryza sativa*; Sl, *Solanum lycopersicum*; Sme, *Solanum melongena*; Solyc, *Solanum lycopersicum*; Sotub, *Solanum tuberosum*

Four putative genes were identified in the 28.5 kb region that contained the *ms32* locus by searching the tomato genome annotation database (ITAG release 3.2) in the Sol Genomics Network (SGN, https://solgenomics.net/; Fig. 3d and Table 1). qPCR analysis revealed that *Solyc01g081090* and *Solyc01g081100* were highly expressed in stamens (Fig. S3). However, the expression of *Solyc01g081080* was barely detected in any organs analyzed in this study (Fig. S3), and the expression of *Solyc01g081110* in sepals and petals was higher than that in stamens and carpels (Fig. S3). The genes *Solyc01g081090* and *Solyc01g081100* encoded bHLH transcription factors. Phylogenic analysis showed that they were within the same clade, which also included *AtbHLH10/89/91*, *OsETA1*, and *OsTIP2* (Fig. 4), which play important roles in pollen and tapetum development^17,18,27^. Therefore, *Solyc01g081090* and *Solyc01g081100* seemed to be candidate genes for *ms32*. Sequencing results showed that there was no sequence difference between the WT and *ms32* mutant within *Solyc01g081090*. However, there was a SNP from G to A in the coding region of *Solyc01g081100* (Fig. 3e), which resulted in a premature stop codon and protein truncation (Fig. 3e and S4). Therefore, *Solyc01g081100* was the most likely candidate gene of *MS32*.

**Table 1:** Predicted genes in the *ms32* region

ID	Gene name^a^	Position on SL3.0ch01	Putative function
1	Solyc01g081080.2.1	80273359-80281965 (−)	Replication factor C subunit
2	Solyc01g081090.2.1	80284835-80287067 (−)	bHLH transcription factor 003
3	Solyc01g081100.2.1	80290985-80292911 (−)	bHLH transcription factor 004
4	Solyc01g081110.3.1	80284835-80300423 (−)	Pentatricopeptide repeat-containing protein

^a^Genes were identified by using the tomato gene model (ITAG release 3.2, SL3.0) in the SGN (https://solgenomics.net/)

### Expression analysis of the genes involved in pollen and tapetum development

**Fig. 5:**
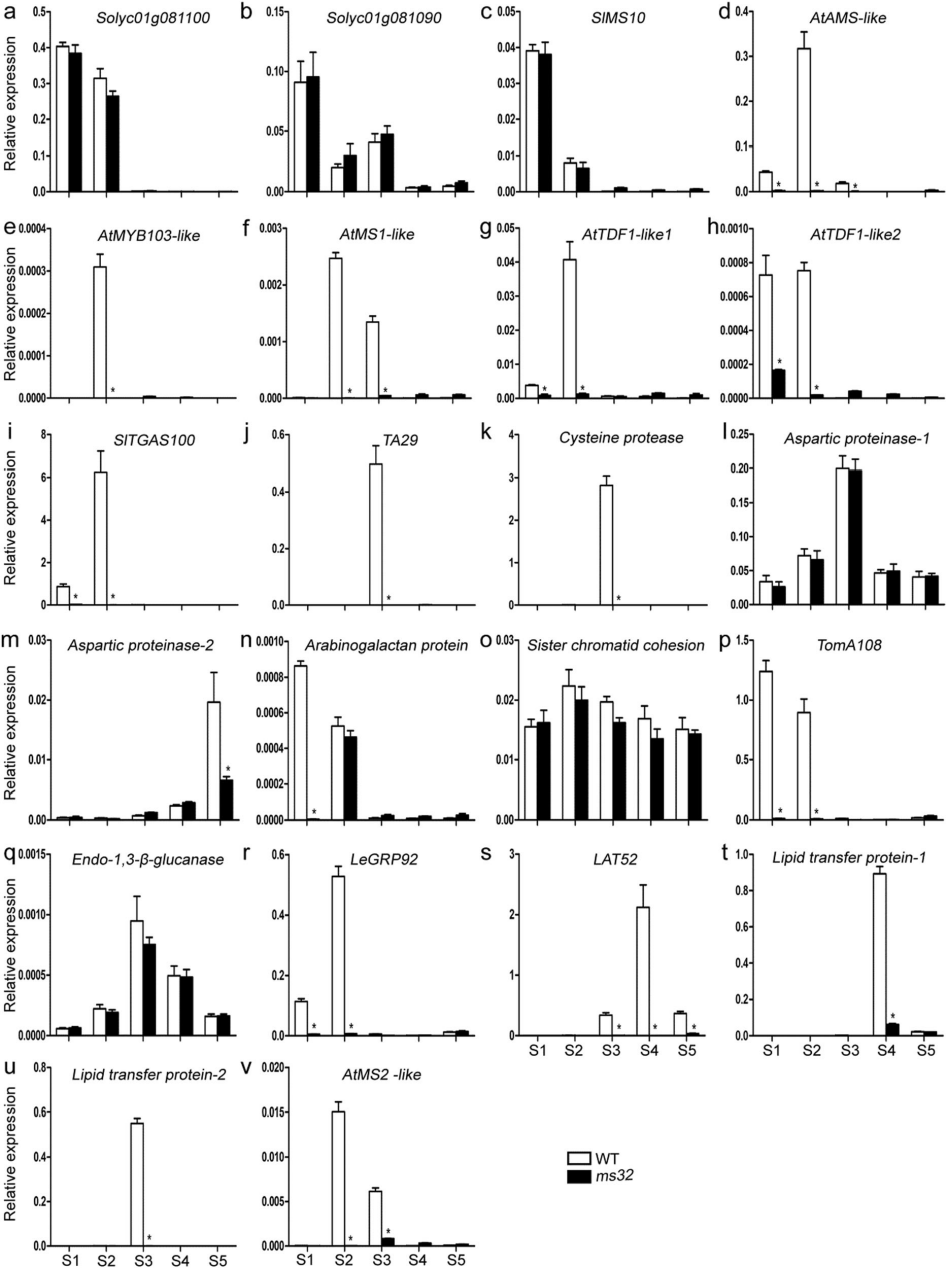
Transcription/expression of the genes related to pollen and tapetum development in flower buds of WT and *ms32* plants. qPCR of the *Solyc01g081100* (**a**), *Solyc01g081090* (**b**), *SlMS10* (**c**), *AtAMS-like* (**d**), *AtMYB103-like* (**e**), *AtMS1-like* (**f**), *AtTDF1-like1* (**g**), *AtTDF1-like2* (**h**), *SlGAS100* (**i**), *TA29* (**j**), *cysteine protease* (**k**), *aspartic protease-1* (**l**), *aspartic protease-2* (**m**), *arabinogalactan protein* (**n**), *sister chromatid cohesion* (**o**), *TomA108* (**p**), *endo-1,3-β-glucanase* (**q**), *LeGRP92* (**r**), *LAT52* (**s**), *lipid transfer protein-1* (**t**), *lipid transfer protein-1* (**u**), and *AtMS2-like* (**v**) genes. Values represent the means ± SEs. Asterisks indicate a significant difference (*p* < 0.05) between WT and *ms32* plants. S1–S5, flower buds at five stages of anther development. S1, meiosis and tetrad stage; S2, microspore stage; S3, mitotic stage; S4, dehiscence stage; S5, opened flower stage

Given that the *ms32* mutant exhibited abnormal pollen and tapetum development (Fig. 2) as well as contained a SNP that was predicted to cause a premature stop codon in the *Solyc01g081100* gene (Fig. 3e and S4), which is a homolog of the pollen and tapetum development regulator genes *AtbHLH10/89/91*, *OsETA1*, and *OsTIP2* (Fig. 4)^17,18,27^, the transcriptional expression of several genes previously proposed to be related to tomato pollen and tapetum development were detected in flowers of WT and *ms32* plants^24,32^. For the genes encoding transcription factors, *Solyc01g081090, Solyc01g081100*, and *SlMS10* (*Solyc02g079810*) were expressed highly and almost equally in the early stages of flower development in WT and *ms32* plants (Fig. 5a–c). In contrast, *AtAMS-like* (*Solyc08g062780*), *AtTDF1-like1* (*Solyc03g113530*), *AtTDF1-like2* (*Solyc03g059200*), *AtMS1-like* (*Solyc04g008420*), and *AtMYB103-like* (*Solyc10g005760*) showed significant downregulation in the *ms32* mutant (Fig. 5d–h).

For the genes related to tapetum degradation, *Cysteine protease* (*Solyc07g053460*), *TA29* (*Solyc02g078370*)^33^, and *TGAS100* (*Solyc06g064470*)^34^ were downregulated in the *ms32* mutant (Fig. 5i–k); *Arabinogalactan protein* (*Solyc11g072780*) and *Aspartic proteinase-2* (*Solyc08g068870*) were only significantly downregulated at the earliest and latest stages of flower development in the *ms32* mutant, respectively (Fig. 5m, n). However, *Aspartic proteinase-1* (*Solyc06g069220*) was expressed almost equally between WT and *ms32* flowers in all stages examined (Fig. 5l). For the genes associated with pollen formation and maturation, *TomA108* (*Solyc01g009590*)^35^, *Lipid transfer protein-1* (*Solyc06g059790*), *Lipid transfer protein-2* (*Solyc01g095780*), *AtMS2-like* (*Solyc03g051960*), *LAT52* (*Solyc10g007270*)^36,37^, and *LeGRP92* (*Solyc02g032910*)^38^ showed downregulation in the *ms32* mutant (Fig. 5p, r–v). In contrast, *Sister chromatid cohesion* (*Solyc03g116930*) and *Endo-1,3-β-glucanase* (*Solyc03g046200*) were expressed almost equally between WT and *ms32* flowers in all stages examined (Fig. 5o, q).

## Discussion

### *Solyc08g091100*, a putative bHLH transcription factor gene, is a candidate gene for the *ms32* locus controlling male sterility in tomato

The tomato *ms32* mutant is considered useful in hybrid seed production, mainly due to the following advantages: (1) stably and completely sterile and (2) accessible for pollination without emasculation due to the exerted stigma^3^. However, *ms32* is a recessive mutation, and its mutant phenotype is expressed at the flowering stage (Fig. 1); thus, it is inconvenient to distinguish fertile and sterile plants when the *ms32* line is used in hybrid seed production and the *ms32* locus is introduced into the inbred line. The availability of molecular markers tightly linked to the *MS32* mutant gene would facilitate functional studies and breeding. In this study, the *ms32* locus was fine mapped to a 28.5 kb interval that encoded four putative genes (Fig. 3). *Solyc01g081100* contained a SNP in the coding region in the *ms32* mutant, which resulted in a premature stop codon and protein truncation (Fig. 3e and S4). *Solyc01g081100* is a homolog of *AtbHLH10/89/91*, *OsETA1*, and *OsTIP2* (Fig. S4), whose loss-of-function mutants are also male sterile^17,18,27^. However, no sequence differences and no expression differences were detected between the WT and *ms32* mutant for the genes *Solyc01g081080*, *Solyc01g081090*, and *Solyc01g081110* (Fig. 5b and S5). Therefore, *Solyc01g081100* was the most likely candidate gene of *MS32*. A *MS32* candidate gene-specific marker, MS32D, was developed based on the SNP in the coding region of *Solyc01g081100*. MS32D successfully distinguished the homozygous WT, heterozygote, and homozygous *ms32* mutant (Fig. S6). Therefore, this marker may facilitate MAS of *ms32* in the breeding of tomato male-sterile female parents in hybrid seed production.

### The *MS32* gene regulates pollen and tapetum development

The tomato *ms32* mutant did not release pollen from the flowers at anthesis (Fig. 1). Histological examination revealed defects in microgametogenesis and tapetum degradation resulting from the *ms32* mutation (Fig. 2). At and after the tetrad stage, in *ms32* anthers, PMCs were degenerated and failed to successively produce tetrads, microspores, and pollen grains. Additionally, tapetal cells were abnormally enlarged and vacuolated without degeneration (Fig. 2). Fine-mapping and sequencing results indicated that *Solyc01g081100*, a homolog of *AtbHLH10/89/91* and *OsEAT1* that regulates pollen and tapetum development, was the most likely candidate gene of *MS32* (Figs. 3 and 4). qPCR also showed that most of the genes proposed to be involved in pollen and tapetum development in tomato were downregulated in the *ms32* mutant (Fig. 5). These findings suggest that the *MS32* gene is involved in the regulation of pollen and tapetum development.

Previous studies have suggested that Arabidopsis and rice share a highly conserved regulatory network of pollen and tapetum development^23^. Two pathways, namely, the *DYT1*-*TDF1*-*AMS*-*bHLH10/89/91*-*MYB80* transcriptional cascade in Arabidopsis and the *UDT1*-*TDR1*-*TIP2*-*EAT1* transcriptional cascade in rice, have been suggested to regulate pollen and tapetum development^17,18,23,24^. Recently, *SlMS10* (*Solyc02g079810*), a homolog of *AtDYT1* and *OsUDT1*, has been proposed to lead a similar transcriptional cascade regulating pollen and tapetum development in tomato^24^. Loss of function of the *SlMS10* gene results in downregulation of the transcription factor genes *AtTDF1-like*, *AtAMS*-*like*, *AtbHLH89/91*-*like* (*Solyc01g081100*), *AtMYB103*-*like*, and *AtMS1*-*like*^24^. In this study, most of these genes were downregulated in the *ms32* mutant, except the *AtbHLH89/91*-like genes (*Solyc01g081090* and *Solyc01g081100*) and *SlMS10* (Fig. 5a–h). Based on the fine-mapping and sequencing results in this study and transcription analysis results in this and previous studies^24^, *Solyc01g081100* is proposed to be the candidate gene of *MS32* and to be a regulator gene downstream of *SlMS10*. The results also suggested that there was no self-feedback regulation of *Solyc01g081100* expression and no feedback regulation between the *MS32* and *SlMS10* genes. However, *SlMS10* and *MS32* might work together to regulate pollen and tapetum development in tomato. In Arabidopsis, AtDYT1 upregulates the expression of *AtbHLH89* and forms a protein complex with AtbHLH89 protein to activate the expression of *AtTDF1*^22^. Similarly, in this and previous studies^24^, mutations in *SlMS10* and *Solyc01g081100* were related to the downregulation of *AtTDF1*-*like* genes (Fig. 5g, h). It is necessary to detect whether SlMS10 interacts with Solyc01g081100 to promote the expression of *AtTDF1*-*like* genes.

The *AtbHLH10*, *AtbHLH89*, and *AtbHLH91* genes resulted from duplication and are functionally redundant for normal pollen and tapetum development in Arabidopsis. Single mutants in each of the *AtbHLH10*, *AtbHLH89,* and *AtbHLH91* genes are developmentally normal and without obviously defective pollen grains^17^. Two *AtbHLH89/91*-*like* genes, namely, *Solyc01g081090* and *Solyc01g081100*, were identified in tomato. These two genes might be the result of tandem duplication as they are closely linked on the same chromosome. This tandem duplication might have occurred before the separation of *Solanaceae* species because potato, pepper, and eggplant all contain two *AtbHLH89/91*-*like* genes that are tightly linked on the same chromosome (Fig. 4). The tomato *ms32* mutant was completely male sterile (Fig. 1 and S1). It contained a mutation in *Solyc01g081100*, while there was no sequence polymorphism or expression difference in *Solyc01g081090* (Figs. 3 and 5b), which suggested functional divergence of these two genes. In rice, *OsEAT1*, a homolog of *Solyc01g081090* and *Solyc01g081100*, activates tapetal cell death by regulating aspartic proteases^27^. An *aspartic protease* gene (*Solyc06g069220*) was also found to be downregulated in the tomato *ms10*^*35*^ mutant and the *SlMED18* RNA interference line, which are both defective in tapetum development^24,32^. However, this *aspartic protease* gene did not show significantly different expression in the tomato *ms32* mutant (Fig. 5l). Furthermore, *Solyc01g081100*, the candidate gene of *MS32*, is downregulated in the tomato *ms10*^*35*^ mutant but not in the *SlMED18* RNA interference line. In contrast, *SlMS10*, putatively encoding a regulator upstream of *Solyc01g081100* in the transcriptional cascade in tomato, is downregulated in both the tomato *ms10*^*35*^ mutant and the *SlMED18* RNA interference line^24,32^. These findings suggest that the other bHLH transcription factor, in addition to *Solyc01g081100*, might also be involved in the regulation of tapetum development. Further studies on *Solyc01g081090* and *Solyc01g081100* may help us understand the details of pollen and tapetum development regulation in tomato.

In summary, in this study, the tomato *ms32* mutant did not release pollen and exhibited abnormalities in pollen and tapetum development (Figs. 1 and 2). The *ms32* locus was fine mapped to a 28.5 kb interval that encoded four putative genes. *Solyc01g081100* was proposed to be the candidate gene of *MS32* as it contained a SNP that resulted in the formation of a premature stop codon (Fig. 3 and S4). The candidate gene-specific marker MS32D (Fig. S6), which was developed according to the SNP in the gene *Solyc01g081100*, can be used for MAS of male-sterile inbred lines of tomato. *Solyc01g081100* can also be used as the target for gene editing to quickly develop such inbred lines. qPCR analysis showed that the expression levels of several genes involved in pollen and tapetum development were changed in the *ms32* mutant (Fig. 5). These findings will support studies on the regulatory mechanisms of pollen and tapetum development in tomato.

## Materials and methods

### Plant materials

Seeds of *ms32* (TGRC accession number LA2714, background genotype: Montfavet-168) and LA1589, the wild tomato species *Solanum pimpinellifolium*, were obtained from the Tomato Genetics Resource Center (TGRC, Davis, CA, USA). The source of the other seeds used in this study was previously described^39^. The male-sterile plant and homozygous male-fertile plant derived from LA2714 were named the *ms32* mutant and WT, respectively. A total of 665 plants of an F_2_ population derived from a cross between the *ms32* mutant and the wild tomato species LA1589 were grown in an open field in Shunyi District (Beijing, China) in the spring and summer of 2016. For the phenotypic analysis and expression analysis, the WT and *ms32* mutant plants were grown in a greenhouse in Haidian District (Beijing, China) in the spring and summer of 2018.

### Phenotypic analysis and histological examination

The morphology of the WT and *ms32* mutant flowers was observed at anthesis. The entire flower was photographed by a camera (Canon EOS 70D, Canon Inc., Japan). Pollen viability was tested using the method described by Sinha and Rajam^40^. Histological examination was performed using the method described by Brukhin et al.^41^. Slides were observed under a microscope (Olympus BX51TRF, Olympus Corporation, Tokyo, Japan).

### Molecular marker development and genotyping

InDels were identified by comparing the sequences of chromosome 1 of tomato lines Heinz 1706 and LA1589, whose whole-genome sequence was published and released in the SGN (https://solgenomics.net/)^42^. PCR primers matching the flanking regions of these InDels were designed using the Primer-BLAST tool available from the National Center for Biotechnology Information (NCBI, http://www.ncbi.nlm.nih.gov/tools/primer-blast/). These primers were used to test for polymorphisms between the *ms32* mutant and LA1589. The *MS32* candidate gene-specific marker MS32D was developed based on DNA polymorphism within the *MS32* candidate gene. General information regarding the DNA markers used for fine mapping of *ms32* is given in Table S1. The PCR conditions were as follows: 94 °C for 4 min; followed by 35 cycles of 94 °C for 30 s, 55 °C for 30 s, and 72 °C for 30–60 s; and 72 °C for 5 min. The PCRs were performed on a PCR instrument (Bio-Rad, Hercules, CA, USA), and the PCR products of these markers were separated on a 3.0% agarose gel. For the marker MS32D, the PCR products were digested with 2 U DdeI restriction enzyme for 2 h, after which the digested products were separated on a 3% agarose gel.

### Genetic analysis and fine mapping

The segregation ratio of the *ms32* locus was tested by chi-square analysis using 665 individuals of an F_2_ population. For preliminary mapping of the *ms32* locus, 94 sterile plants in the F_2_ population were genotyped using six InDel markers in the interval between genes *Y* (*SlMYB12*) and *Cf-4* on chromosome 1, as the *ms32* locus is located in this interval in the genetic map reported in 1987^29–31^. For fine mapping of the *ms32* locus, the whole F2 population was genotyped using markers HP4547 and HP1693. A total of 13 recombinants, which were determined to be homozygous for the *ms32* mutant for just one of these two markers, were selected for further genotyping using additional markers (see Table S1).

### Gene prediction

The putative genes in the fine-mapping region harboring the *ms32* locus were identified using the tomato gene model (ITAG release 3.2) in the SGN (https://solgenomics.net).

### Phylogenetic tree construction

The protein sequences of the known bHLH transcription factors *AtAMS*, *AtbHLH10*, *AtbHLH89*, *AtbHLH91*, *AtDYT1*, *OsEAT1*, *OsTDR1*, *OsTIP2*, and *OsUDT1*, which are related to pollen and tapetum development in Arabidopsis and rice, were downloaded from The Arabidopsis Information Resource (https://www.arabidopsis.org/) and GenBank. Using the full-length protein sequences of these bHLH transcription factors as queries, the homologs of eggplant, pepper, potato and tomato were identified by BLASTP in the databases of eggplant genome protein sequences (release 2.5), *Capsicum annuum* cv CM334 genome protein sequences (release 1.55), *Capsicum annuum* var. glabriusculum genome protein sequences (v2.0), *Capsicum annuum* var. zunla genome protein sequences (v2.0), potato ITAG Release 1 predicted proteins (ST1.0), and tomato genome proteins (ITAG release 3.20) in the SGN with an *E*-value ≤ 1 × 10^−50^. Multiple alignment of the full-length sequences of these bHLH transcription factors was performed by Clustal X ver. 2.1^43^ using the default settings. An unrooted phylogenetic tree was constructed by using MEGA6^44^ with the neighbor-joining method, Jones-Taylor-Thornton (JTT) model, and 1000 replicates.

### Sequence polymorphism analysis

The genomic fragments of the candidate genes of *MS32*, namely, *Solyc01g081090* and *Solyc01g081100*, were obtained from the WT and *ms32* mutant by overlapping PCR. The amplified fragments were sequenced at the Beijing Genomics Institute (Beijing, China). The partial cDNAs of the *Solyc01g081100* gene were obtained by reverse-transcription PCR (RT-PCR), which was performed using Phusion High-Fidelity DNA Polymerase (Cat. No. M0530L; New England Biolabs, Ipswich, MA, USA) and the primers for cDNA cloning (Table S1). The amplified fragments were cloned using a pEASY-Blunt Zero Cloning Kit (Cat. No. CB501-02; TransGen Biotech, Beijing, China). The cDNA clones were sequenced at the Beijing Genomics Institute.

### RNA extraction, cDNA synthesis, and qPCR analysis

Flower buds at different developmental stages were collected from WT and *ms32* plants and rapidly frozen in liquid nitrogen. Each tissue sample comprised three biological replicates, and each replicate contained samples from at least three plants. Total RNA was isolated using the Quick RNA Isolation Kit (Cat. No. BC1803, Huayueyang Biotech Co. Ltd., Beijing, China), and cDNA was synthesized from 2 μg total RNA using GoScript Reverse Transcriptase (Cat. No. A5003; Promega, Madison, WI, USA). The qPCRs were conducted using GoScript^TM^ qPCR Master Mix (Cat. No. A6002; Promega) and a LightCycler 480 Detection System (Roche Diagnostics GmbH, Mannheim, Germany). The primers for qPCR are provided in Table S1. qPCR was performed using the methods described previously by Cao et al.^45^.

## Supplementary information

Tomato ms32 supplementary figures and tables

## References

[1] Bai YL, Lindhout P (2007). Domestication and breeding of tomatoes: What have we gained and what can we gain in the future?. Ann. Bot.-Lond..

[2] Tamta S, Singh JP (2018). Heterosis in tomato for growth and yield traits. Int. J. Veg. Sci..

[3] Atanassova, B. Genic male sterility and its application in tomato (*Lycopersicon esculentum* mill.) hybrid breeding and hybrid seed production. *Proceedings of the IIIrd Balkan Symposium on Vegetable and Potatoes*, 45–51 (2007).

[4] Kaul, M. L. H. *Male sterility in higher plants* (Springer, Berlin, 1988).

[5] Gómez Pedro, Jamilena Manuel, Capel Juan, Zurita Sergio, Angosto Trinidad, Lozano Rafael (1999). Stamenless , a tomato mutant with homeotic conversions in petals and stamens. Planta.

[6] Sawhney VK, Greyson RI (1973). Morphogenesis of the stamenless-2 mutant in tomato. I. Comparative description of the flowers and ontogeny of stamens in the normal and mutant plants. Am. J. Bot..

[7] Larson, R. E. & Paur, S. The description and the inheritance of a functionally sterile flower mutant in tomato and its probable value in hybrid tomato seed production. *Proc. Am. Soc. Hort. Sci*. **52**, 355–364 (1948).

[8] Atanassova B (1999). Functional male sterility (*ps-2*) in tomato (*Lycopesicon esculentum* Mill.) and its application in breeding and hybrid seed production. Euphytica.

[9] Rick CM, Robinson J (1951). Inherited defects of floral structure affecting fruitfulness in *Lycopersicon esculentum*. Am. J. Bot..

[10] Gorman SW, McCormick S, Rick C (1997). Male Sterility in Tomato. Crit. Rev. Plant Sci..

[11] Wilson ZA, Zhang DB (2009). From Arabidopsis to rice: pathways in pollen development. J. Exp. Bot..

[12] Phan HA, Iacuone S, Li SF, Parish RW (2011). The MYB80 transcription factor is required for pollen development and the regulation of tapetal programmed cell death in Arabidopsis thaliana. Plant Cell.

[13] Sorensen AM (2003). The *Arabidopsis ABORTED MICROSPORES (AMS)* gene encodes a MYC class transcription factor. Plant J.

[14] Zhang W. (2006). Regulation of Arabidopsis tapetum development and function by DYSFUNCTIONAL TAPETUM1 (DYT1) encoding a putative bHLH transcription factor. Development.

[15] Zhang Zai-Bao, Zhu Jun, Gao Ju-Fang, Wang Chen, Li Hui, Li Hong, Zhang Hui-Qi, Zhang Sen, Wang Dong-Mi, Wang Quan-Xi, Huang Hai, Xia Hui-Jun, Yang Zhong-Nan (2007). Transcription factor AtMYB103 is required for anther development by regulating tapetum development, callose dissolution and exine formation in Arabidopsis. The Plant Journal.

[16] Zhu J (2008). *Defective in Tapetal development and function 1* is essential for anther development and tapetal function for microspore maturation in Arabidopsis. Plant J..

[17] Zhu E (2015). The DYT1-interacting proteins bHLH010, bHLH089 and bHLH091 are redundantly required for Arabidopsis anther development and transcriptome. Plant J..

[18] Fu Z (2014). The Rice Basic Helix-Loop-Helix Transcription Factor TDR INTERACTING PROTEIN2 Is a Central Switch in Early Anther Development. Plant Cell.

[19] Zhu J, Lou Y, Xu X, Yang ZN (2011). A genetic pathway for tapetum development and function in Arabidopsis. J. Integr. Plant Biol..

[20] Xu, J. et al. The *ABORTED MICROSPORES* regulatory network is required for postmeiotic male reproductive development in *Arabidopsis thaliana*. *Plant Cell* **22**, 91–107 (2010). 10.1105/tpc.109.071803 PMC2828693 20118226

[21] Ferguson AC (2017). Biphasic regulation of the transcription factor ABORTED MICROSPORES (AMS) is essential for tapetum and pollen development in Arabidopsis. New Phytol.

[22] Cui J (2016). Feedback Regulation of DYT1 by Interactions with Downstream bHLH Factors Promotes DYT1 Nuclear Localization and Anther Development. Plant Cell.

[23] Gomez JF, Talle B, Wilson ZA (2015). Anther and pollen development: A conserved developmental pathway. J. Integr. Plant Biol..

[24] Jeong HJ (2014). Tomato *Male sterile 10*^*35*^ is essential for pollen development and meiosis in anthers. J. Exp. Bot..

[25] Jung KH (2005). Rice *Undeveloped Tapetum1* is a major regulator of early tapetum development. Plant Cell.

[26] Li N (2006). The rice *tapetum degeneration retardation* gene is required for tapetum degradation and anther development. Plant Cell.

[27] Niu N (2013). EAT1 promotes tapetal cell death by regulating aspartic proteases during male reproductive development in rice. Nat. commun..

[28] Rick CM (1960). New male-sterile mutants. Rep. Tomato Genet Coop..

[29] Mutschler, M. A., Rick, C. M. & Tanksley, S. D. Linkage maps of the tomato (*Lycopersicon esculentum*). *Rep. Tomato Genet Coop.* **37**, 5–34 (1987).

[30] Lin T (2014). Genomic analyses provide insights into the history of tomato breeding. Nat. Genet.

[31] Parniske Martin, Hammond-Kosack Kim E, Golstein Catherine, Thomas Colwyn M, Jones David A, Harrison Kate, Wulff Brande B.H, Jones Jonathan D.G (1997). Novel Disease Resistance Specificities Result from Sequence Exchange between Tandemly Repeated Genes at the Cf-4/9 Locus of Tomato. Cell.

[32] Perez-Martin F (2018). Developmental role of the tomato Mediator complex subunit MED18 in pollen ontogeny. Plant J..

[33] Nawaz-ul-Rehman, M. S., Mansoor, S., Khan, A. A., Zafar, Y. & Briddon, R. W. RNAi-mediated male sterility of tobacco by silencing *TA29*. *Mol. Biotechnol.* **36**, 159–165 (2007). 10.1007/s12033-007-0025-1 17914195

[34] Van den Heuvel, K. J., Van Lipzig, R. H., Barendse, G. W. & Wullems, G. J. Regulation of expression of two novel flower-specific genes from tomato (*Solanum lycopersicum*) by gibberellin. *J. Exp. Bot.* **53**, 51–59 (2002). 10.1093/jexbot/53.366.51 11741041

[35] Chen RD, Zimmermann E, Xu SX, Liu GS, Smith AG (2006). Characterization of an anther- and tapetum-specific gene and its highly specific promoter isolated from tomato. Plant cell rep..

[36] Twell D, Wing R, Yamaguchi J, McCormick S (1989). Isolation and expression of an anther-specific gene from tomato. Mol. Gen. Genet.

[37] Muschietti, J., Dircks, L., Vancanneyt, G. & McCormick, S. LAT52 protein is essential for tomato pollen development: pollen expressing antisense *LAT52* RNA hydrates and germinates abnormally and cannot achieve fertilization. *Plant J.* **6**, 321–338 (1994). 10.1046/j.1365-313x.1994.06030321.x 7920720

[38] McNeil KJ, Smith AG (2010). A glycine-rich protein that facilitates exine formation during tomato pollen development. Planta.

[39] Zhang L (2016). Fine mapping and molecular marker development of *anthocyanin absent*, a seedling morphological marker for the selection of *male sterile 10* in tomato. Mol. Breed..

[40] Sinha R, Rajam MV (2013). RNAi silencing of three homologues of S-adenosylmethionine decarboxylase gene in tapetal tissue of tomato results in male sterility. Plant Mol. Biol..

[41] Brukhin, V., Hernould, M., Gonzalez, N., Chevalier, C. & Mouras, A. Flower development schedule in tomato *Lycopersicon esculentum* cv. sweet cherry. *Sex. Plant Reprod.* **15**, 311–320 (2003).

[42] Sato S (2012). The tomato genome sequence provides insights into fleshy fruit evolution. Nature.

[43] Larkin MA (2007). Clustal W and Clustal X version 2.0. Bioinformatics.

[44] Tamura K, Stecher G, Peterson D, Filipski A, Kumar S (2013). MEGA6: molecular evolutionary genetics analysis version 6.0. Mol. Biol. Evol..

[45] Cao Xue, Qiu Zhengkun, Wang Xiaotian, Van Giang Tong, Liu Xiaolin, Wang Jing, Wang Xiaoxuan, Gao Jianchang, Guo Yanmei, Du Yongchen, Wang Guoping, Huang Zejun (2017). A putative R3 MYB repressor is the candidate gene underlying atroviolacium, a locus for anthocyanin pigmentation in tomato fruit. Journal of Experimental Botany.

