# Supplementary material for: A putative bHLH transcription factor is a candidate gene for male sterile 32, a locus affecting pollen and tapetum development in tomato
Source: Hortic Res. 2019 Jul 21;6:88. doi: 10.1038/s41438-019-0170-2 (PMC6804878; doi:10.1038/s41438-019-0170-2)
Supplement: Supplementary file 1 — Tomato ms32 supplementary figures and tables [file 41438_2019_170_MOESM1_ESM.pdf]

A putative bHLH transcription factor is a candidate gene for *male sterile 32*, a locus affecting pollen and tapetum development in tomato

Xiaoyan Liu[1, 2], Mengxia Yang[2], Xiaolin Liu[2], Kai Wei[2], Xue Cao[2], Xiaotian Wang[2], Xiaoxuan Wang[2], Yanmei Guo[2], Yongchen Du[2], Junming Li[2], Lei Liu[2], Jinshuai Shu[2], Yong Qin[1, *], Zejun Huang[2, *]

1 College of Forestry and Horticulture, Xinjiang Agricultural University, Urumqi 830052, China

2 Key Laboratory of Biology and Genetic Improvement of Horticultural Crops of the Ministry of Agriculture, Institute of Vegetables and Flowers, Chinese Academy of Agricultural Sciences, Beijing 100086, China

* Corresponding author, Yong Qin, E-mail: 352167610@qq.com, Tel: 86-991-8762361; Zejun Huang, E-mail: huangzejun@caas.cn, Tel: 86-10-82109530

Supplementary Figures and Tables

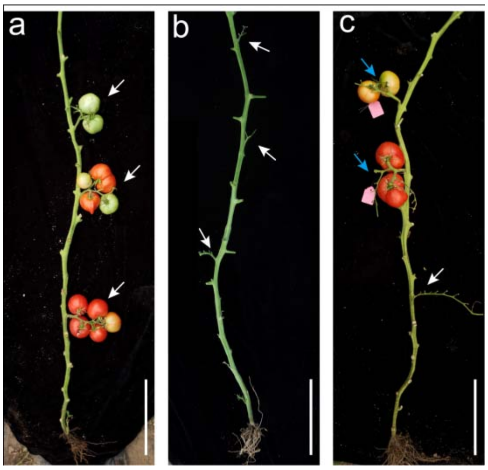

WT ***ms32*** ***ms32***

Figure S1 Fruit set of *ms32* plants. White arrows indicate self-cross and blue arrows indicate out-cross. Scale bars, 20 cm.

```
AtbHLH089                      ----------------------------MGGGGMFEEIGCFDPNAPAEMTAESSFSPSE-   31
AtbHLH010                      -------------------------------------MGCFDPNTPAEVTVESSFSQAE-   22
AtbHLH091                      ---------------------------------MYEESSCFDPNSMVDNNGGFCAAETT-   26
Solyc01g081090.2               --------------------------------MYASQSLSFHPTSD---QED--EKSFV-   22
Sotub01g028050.1.1             --------------------------------MYASQSLSFHPTSDH--QQD--EKSFL-   23
Sme2.5_00114.1_g00006.1        --------------------------------MYASESLSFDPTSS---QQD--EKGFL-   22
CA01g26780                     --------------------------------MYAGESLSFDPTSQQNEVLD--EKGLL-   25
Capang08g000242                --------------------------------MYAGESLSFDPTSQQNEVLD--EKGLL-   25
Solyc01g081100.2               --------------------------------MYVEESVCYDPATHHVQHEG-LTEDVF-   26
Sotub01g028040.1.1             --------------------------------MYVEESVCYDPATHHVQHEG-LTEDVF-   26
Sme2.5_00114.1_g00007.1        --------------------------------MYVEESACFDPAT-HVQHEG-LAEDVF-   25
CA01g26770                     ----------MTSYVLFFFNRSTKVLQQKTKKMYVEESGCFDADTHHVQNDGVLAEDVF-   49
Capana01g003305                --------------------------------MYVEESGCFDADTHHVQNDGVLAEDVF-   27
Capana00g000514                --------------------------------MYVEESGCFDAATHHVQNDGVLAEDVF-   27
Capang08g000243                --------------------------------MYVEESGCFDAATHHVQNDGVLAEDVF-   27
OsEAT1                         ---------------------------------MIVGAGYFEDSHDQSLMAGSLIHDSN-   26
Sotub02g022820.1.1             ------------------------------------------------------------    -
Sme2.5_05438.1_g00004.1        ------------------------------------------------------------    -
SlMS10                         ------------------------------------------------------------    -
Capang02g001895                ------------------------------------------------------------    -
Capana02g002096                ------------------------------------------------------------    -
CA02g17520                     ------------------------------------------------------------    -
AtDYT1                         ------------------------------------------------------------    -
Solyc08g062780.2               ----------------------MELMHLMERLRPIMSLKGWDYCVLWKLSEDQRFLEWIC   38
Sotub08g012880.1.1             ----------------------------MERLRPIMGLKSWDYCVLWKLSEDQRFLEWIC   32
Capang00g000803                ----------------------MELMHLMDRLRPVMGLKSWDYCVLWKLSEDQRFLEWIC   38
Capana08g000254                ----------------------MELMHLMDRLRPVMGLKSWDYCVLWKLSEDQRFLEWIC   38
CA08g01690                     ----------------------MELMHLMDRLRPVMGLKSWDYCVLWKLSEDQRFLEWIC   38
AtAMS                          --------------------MESNMQNLLEKLRPLVGARAWDYCVLWRLNEDQRFVKWMG   40
OsTDR                          MGRGDHLLMKNSNAAAAAAAVNGGGTSLDAALRPLVGSDGWDYCIYWRLSPDQRFLEMTG   60
OsTIP2                         ---------------------------------------MYHPQCELLMPLESLEMDVG-   20
OsUDT1                         ------------------------------------------------------------    -


AtbHLH089                      --------------------------------PPP---TITVIGSNSNS-NCS--LEDLS   53
AtbHLH010                      --------------------------------PPPPPPQVLVAGSTSNS-NCSVEVEELS   49
AtbHLH091                      --------------------------------FTVSHQFQPPLGSTTNSFDDDLKLPTMD   54
Solyc01g081090.2               --------------------------------QNHIAASG----FVELQQQQQ-------   39
Sotub01g028050.1.1             --------------------------------QNHIATSG----LVELQQQ---------   38
Sme2.5_00114.1_g00006.1        --------------------------------QNHIATTSNTSFPMELQQQ---------   41
CA01g26780                     --------------------------------QNHIPTTCVTNFSMGEELQQ--------   45
Capang08g000242                --------------------------------QNHIPITCVTNFSMGEELQQ--------   45
Solyc01g081100.2               --------------------------------VIQE-HTYHNN-NDSSQQDVAVAAAAAA   52
Sotub01g028040.1.1             --------------------------------AIQE-HTYHNN-NNSSQQDVAAAAAAAA   52
```

```
Sme2.5_00114.1_g00007.1      --------------------------------AIQE-HTYHNNSNNSSQQDVA---AAAA   49
CA01g26770                   --------------------------------AIQEQHTYHHN---PSQQDVA----AAA   70
Capana01g003305              --------------------------------AIQEQHTYHHN---PSQQDVA----AAA   48
Capana00g000514              --------------------------------AIQEQHTYHHN---PSQQDVA----AAA   48
Capang08g000243              --------------------------------AIQEQHTYHHN---PSQQDVA----AAA   48
OsEAT1                       --------------------------------QAPASSENTSIDLQKFKVHPYSTEALSN   54
Sotub02g022820.1.1           ------------------------------------------------------------    -
Sme2.5_05438.1_g00004.1      ------------------------------------------------------------    -
SlMS10                       ------------------------------------------------------------    -
Capang02g001895              ------------------------------------------------------------    -
Capana02g002096              ------------------------------------------------------------    -
CA02g17520                   ------------------------------------------------------------    -
AtDYT1                       ------------------------------------------------------------    -
Solyc08g062780.2             CCCGGAEKNMHGCGQEIFFPDSSTSTCRDVMFQHPTTTACNLLAQVPPSLALDCG---VY   95
Sotub08g012880.1.1           CCCGGAEKNMHSCEQEIFFPDSSTSTCRDVMFQHPTTTACDLLAQVPLSLALDCG---VY   89
Capang00g000803              CCCGGADKNMHSCEQELFFPDSSTSTCRDVMFQHPRTIACDLLAQLPPSLALDCG---TY   95
Capana08g000254              CCCGGADKNMHSCEQELFFPDSSTSTCRDVMFQHPRTIACDLLAQLPPSLALDCG---TY   95
CA08g01690                   CCCGGADKNMHSCEQELFFPDSSTSTCRDVMFQHPRTIACDLLAQLPPSLALDCG---TY   95
AtAMS                        CCCGGTELIAENGTEEFSYGG-----CRDVMFHHPRTKSCEFLSHLPASIPLDSG---IY   92
OsTDR                        FCCS-----------------------------SELEAQVSALLDLPSSIPLDSSSIGMH   91
OsTIP2                       ----------------------------------QSHLAAAVAAAMPGELN---------   37
OsUDT1                       ------------------------------------------------------------    -


AtbHLH089                    AFHLSPQDSSLPASASAYAHQLHINATPN-----------------CDHQFQSSMHQTLQ   96
AtbHLH010                    EFHLSPQDC---PQASSTPLQFHINPPPPP-------------PPPCDQLHNNLIHQMA-   92
AtbHLH091                    EFSVFPSVIS---LPNSETQNQNIS------------------------NNNHLINQMIQ   87
Solyc01g081090.2             --QFHSSEN-NLNMQLEFGHDSNNTR------------------------FHPNWEEISF   72
Sotub01g028050.1.1           --QLHSSENMNLNMQLEFGHDPNNTR------------------------FHQNWEEISF   72
Sme2.5_00114.1_g00006.1      --QLNLGESSNLNMQIEFGHEPNNTR------------------------FHPNWEEIGL   75
CA01g26780                   --KFHSSENNNMNMQ-EFGHHDEHTNT-------------------VLPCVHPNWEEIGF   83
Capang08g000242              --KFHSSENNNMNMQ-EFGHHDEHTNT-------------------VLPCVHPNWEEIGF   83
Solyc01g081100.2             -LEIEFQHQLNLEMEQCYNNNNNNNTH----------NNNNNIVNEGLSCDQANWGEMNF  101
Sotub01g028040.1.1           ALEMEFQQQMNLEMEQCYNNNNMQD------------QSNNNIINQGLSCDQSNWGEMNF  100
Sme2.5_00114.1_g00007.1      ALEMEFQQQLNLEMEQCDIDG---------------------------------------   70
CA01g26770                   ALEIEFQQQLNLEMEECYNNTHNNNMQDQLIHDQQNNNNNNN-SNQGLSCDQSNWGEMNF  129
Capana01g003305              ALEIEFQQQLNLEMEECYNNTHNNNMQDQLIHDQQNNNNNNN-SNQGLSCDQSNWGEMNF  107
Capana00g000514              ALEIEFQQQLNLEMEECYNNTHNNNMQDQLIHDQQNNNNNNN-SNQGLSCDQSNWGEMNF  107
Capang08g000243              ALEIEFQQQLNLEMEECYNNTHNNNMQDQLIHDQQNNNNNNNNSNQGLSCDQSNWGEMNF  108
OsEAT1                       TANLAEAARAINHLQHQLEIDLEQEVPPVETAN--------WDPAICTIPDHIINHQFSE  106
Sotub02g022820.1.1           ------------------------------------------------------------    -
Sme2.5_05438.1_g00004.1      ------------------------------------------------------------    -
SlMS10                       ------------------------------------------------------------    -
Capang02g001895              ------------------------------------------------------------    -
```

```
Capana02g002096            ------------------------------------------------------------    -
CA02g17520                 ------------------------------------------------------------    -
AtDYT1                     ------------------------------------------------------------    -
Solyc08g062780.2           AQTLLSNQAKWMNFVPFSESNISNEIMGTRALIPSPLGLLELFSTQQLAEDEKVIEFVSA  155
Sotub08g012880.1.1         AQTLLSNQAKWMNFVSFSESNISNEIMGTRALIPSPLGLLELFSTQQLAEDEKVIEFVSA  149
Capang00g000803            AMALLSNQAKWMNFVPFSISNMTNEIMGTRALVPSPLGLLELFSTQQLPEDEEVIEFVSA  155
Capana08g000254            AMALLSNQAKWMNFVPFSISNMTNEIMGTRALVPSPLGLLELFSTQQLPEDEEVIEFVSA  155
CA08g01690                 AMALLSNQAKWMNFVPFSISNMTNEIMGTRALVPSPLGLLELFSTQQLPEDEEVIEFVSA  155
AtAMS                      AETLLTNQTGWLSES--SEPSFMQETICTRVLIPIPGGLVELFATRHVAEDQNVVDFVMG  150
OsTDR                      AQALLSNQPIWQSSSEEEEADGGGGAK-TRLLVPVAGGLVELFASRYMAEEQQMAELVMA  150
OsTIP2                     -FHLLHSLDAAAAAAASSTAASASSQP---------------------------------   62
OsUDT1                     ------------------------------------------------------------    -

AtbHLH089                  DPSYAQQSNHWDNGYQDFVNLGPN-HTTPD------------------------------  125
AtbHLH010                  --SHQQQHSNWDNGYQDFVNLGPNSATTPD------------------------------  120
AtbHLH091                  E-SNWGVSEDNSNFFMNTSHPNTTTTPIPD------------------------------  116
Solyc01g081090.2           NPYNNQQ---------------LSYPISN-------------------------------   86
Sotub01g028050.1.1         NPYNNQQ---------------LSYPISN-------------------------------   86
Sme2.5_00114.1_g00006.1    NPYNNQQ---------------LSYPISSS------------------------------   90
CA01g26780                 NPNNNQL---------------LSYPISS-------------------------------   97
Capang08g000242            NPNNNQL---------------LSYPISS-------------------------------   97
Solyc01g081100.2           PPYQNQQHNDNGNSNNNFHQQDFSNPISETPYLTTPDL-------------LNMFPLPRC  148
Sotub01g028040.1.1         PPYQNQEHNDNSNNNNNFHQQDFSNPISEPPYLTTPDL-------------LNMFPLPRC  147
Sme2.5_00114.1_g00007.1    ------------------------------------------------------------    -
CA01g26770                 PPYQNQEDSNNINNHNNFQQQNFPIPMSDAPYLTTPDL-------------LNMFPLPRC  176
Capana01g003305            PPYQNQEDSNNINNHNNFQQQNFPIPMSDAPYLTTPDL-------------LNMFPLPRC  154
Capana00g000514            PPYQNQEDSNNINNHNNFQQQNFPIPMSDAPYLTTPDL-------------LNMFPLPRC  154
Capang08g000243            PPYQNQEDSNNINNHNNFQQQNFPIPMSDAPYLTTPDL-------------LNMFPLPRC  155
OsEAT1                     DPQNILVEQQIQQYDSALYPNGVYTPAPDLLN----------------------------  138
Sotub02g022820.1.1         ------------------------------------------------------------    -
Sme2.5_05438.1_g00004.1    ------------------------------------------------------------    -
SlMS10                     ------------------------------------------------------------    -
Capang02g001895            ------------------------------------------------------------    -
Capana02g002096            ------------------------------------------------------------    -
CA02g17520                 ------------------------------------------------------------    -
AtDYT1                     ------------------------------------------------------------    -
Solyc08g062780.2           QCNIYLEQQAMMNSTFSNGVEENNTSKPFPTEGERDRD--DHIKDSQNHYKQRVSPAATS  213
Sotub08g012880.1.1         QCNIYLEQQAMMNSTFSNGVQEN-TSKPFPTEGDGDGDGDDHIKDSQNHYQQTVSPAATS  208
Capang00g000803            QCNIYVEQQAMTNSSFSDGVEES-TSKPFPLEGD------DHIKVSQNHYQQTVSPAATS  208
Capana08g000254            QCNIYVEQQAMTNSSFSDGVEES-TSKPFPLEGD------DHIKVSQNHYQQTVSPAATS  208
CA08g01690                 QCNIYVEQQAMTNSIFSDGVEES-TSKPFPLEGD------DHIKVSQNHYQQTVSPAATS  208
AtAMS                      HCNMLMDDSVTINMMVADEVESK----PYGMLSG-------DIQQKGSKEEDMMN-----  194
OsTDR                      QCGGGGAGDDGGGQAWPPPETPS-----FQWDGG--------------------------  179
```

OsTIP2	--	-
OsUDT1	--	-

AtbHLH089	----------LLSLLQLPRSSLPPFANP------SIQDIIMTTSSSVAAYDPLFHLNFPL	169
AtbHLH010	----------LLSLLHLPRCSLPPNHHPSSMLPTSFSDIMSSSSAAAVMYDPLFHLNFPM	170
AtbHLH091	----------LLSLLHLPRCSMS----------LPSSDIMAG---SCFTYDPLFHLNLPP	153
Solyc01g081090.2	--------PSLGLLGGFHQRT--ELASTSTN-LFYEPPQMNMPLNLCTPQSSLFKELFHL	135
Sotub01g028050.1.1	--------PSN-FLSTTTPSSSLGLLATSTN-LFYEPPQLNMPLNLCTPQSSLFKELFHL	136
Sme2.5_00114.1_g00006.1	--------SSLGLLAGFHQRTDHQLASTSTN-LFYEPPQLYMPLNLCTPQSSLFKELFHL	141
CA01g26780	---------SLGHLGGFHQRT--EIPSTSTN-LFYELPQLNMPINLCTQQPSLFKDLFHL	145
Capang08g000242	---------SLGHLGGFHQRT--EIPSTSTN-LFYEPPQLNMPINLCTQQPSLFKDLFHL	145
Solyc01g081100.2	TQSSLLPQKSPNLLTSLGLIGDIDGGGASTSSAICDPSSLLLPLNLP-PQPPLLRELFHS	207
Sotub01g028040.1.1	SQSSLLPQKSPNLLTSLGLIGDIDGGGASTSTAIYDPSSLVLPLNLP-PQPPLLRELFHS	206
Sme2.5_00114.1_g00007.1	------------------------GGGAPTSSALYDP-SLVLPLNLP-PQPPLLRELFHS	104
CA01g26770	SQSSLLPQKSPNLLTSLELIGDIDGG-ASTSNAVYDP-SLVLPLNLP-PQPPLLRELFHS	233
Capana01g003305	SQSSLLPQKSPNLLTSLELIGDIDGG-ASTSNAVYDP-SLVLPLNLP-PQPPLLRELFHS	211
Capana00g000514	SQSSLLPQKSPNLLTSLELIGDIDGG-ASTSNAVYDP-SLVLPLNLP-PQPPLLRELFHS	211
Capang08g000243	SQSSLLPQKSPNLLTSLELIGDIDGG-ASTSNAVYDP-SLVLPLNLP-PQPPLLRELFHS	212
OsEAT1	---LMQCTMAPAFPATTSVFGDTTLNGTNYLDLNGELTGVAAVPDSGSGLMFASDSALQL	195
Sotub02g022820.1.1	--	-
Sme2.5_05438.1_g00004.1	--	-
SlMS10	--	-
Capang02g001895	--	-
Capana02g002096	--	-
CA02g17520	--	-
AtDYT1	--	-
Solyc08g062780.2	----DHLSFDFPLKRKQLDSCSMNFLPPFSTYSTPEVDNNTGGN-----MLFDQSTSDMT	264
Sotub08g012880.1.1	----DHLSYEFPLKRKQLDTCSMNFLPPFSTYSTPEVDNNTGGN-----MLFNQSTSDAT	259
Capang00g000803	RGHSDNLSYDFPLKRKHLDTSSMNFLPQLSTYSTPEVYNKTGGN-----MLFDQRTSDVT	263
Capana08g000254	RGHSDNLSYDFPLKRKHLDTSSMNFLPQLSTYSTPEVYNKTGGN-----MLFDQRTSDVT	263
CA08g01690	RGHSDNLSYDFPLKRKHLDTSSMNFLPQLSTYSTPEVYNKTGGN-----MLFDQRTSDVT	263
AtAMS	------LPSSYDISADQIR---LNFLPQMSDYETQHLKMKSDYHHQALGYLPENGNKEMM	245
OsTDR	----------ADAQRLMYGGSSLNLFDAAAADDDPFLGGGGGDA------VGDEAAAAGA	223
OsTIP2	-----------TVDYFFGGADQQPPPPAAMQYDQLAAPHHHQTVAMLRDYYGGHYPPAAA	111
OsUDT1	--	-

AtbHLH089	QPP------NGSFMG--VDQDQTE-TNQGVNLMYDE--ENNN---------LDDGLN---	206
AtbHLH010	QPRDQNQLRNGSCLLGVEDQIQMD-ANGGMNVLYFEGANNNNGGFENEILEFNNGVT---	226
AtbHLH091	QPPLIPSNDYSGYLLGIDTNTTTQ-RDE--SNVGDE---NNNAQFDSGIIEFSKEIR---	204
Solyc01g081090.2	SPHGSSYGLGSSGTG--SLFSLGHDQEEVTGNLYHDG--SFH-ELTGDMMINSAAIK---	187
Sotub01g028050.1.1	SPHGSSYGLGSSGTG--SLFSHGHDQEEVTGNLYHDG--SFH-ELSGDMMINSAAIK---	188
Sme2.5_00114.1_g00006.1	PPHGSSYGLGSSWTG--SLFSHGHDQEEAIGSLYHDG--SFH-EFTGDMMN-SAAIK---	192

CA01g26780	SPHGSSYGFDSSGTG--SLFSHGHHEEEVTGALYHDG--SFHHEFSTGDMINSAAIK---	198
Capang08g000242	SPHGSSYGFDSSGTG--SLFSHGHHEEEVTGALYHDG--SFHHEFSTGDMINSAAIK---	198
Solyc01g081100.2	FPHGYGLRNLRSNNN-TSFFNGLEETDQG---LYQEN--GETRPFQNGIFEFSGGMNDIA	261
Sotub01g028040.1.1	FPHGYGLRNLRSNNN-TSFFNGLEEQDQG---LYQEN--GETRPFDNGIFEFSAGMNDIA	260
Sme2.5_00114.1_g00007.1	FPHGYGLRNLRSNNNTTSFFNGLEERDQG---LYQDN--GETRPFDSGIFEFSA----VA	155
CA01g26770	FPHGYGLRNLRSNNN-TSFFNGMEERDQVNGALYQDN--GEGRPFDNGIFEFSAGMNDIA	290
Capana01g003305	FPHGYGLRNLRSNNN-TSFFNGMEERDQVNGALYQDN--GEGRPFDNGIFEFSAGMNDIA	268
Capana00g000514	FPHGYGLRNLRSNNN-TSFFNGMEERDQVNGALYQDN--GEGRPFDNGIFEFSAGMNDIA	268
Capang08g000243	FPHGYGLRNLRSNNN-TSFFNGMEERDQVNGALYQDN--GEGRPFDNGIFEFSTDMNDIA	269
OsEAT1	GYHGTQSHLIKDICHSLPQNYGLFPSEDERDVIIGVGSGDLFQEIDDRQFDSVLECRRGK	255
Sotub02g022820.1.1	-------------------------------MESPNTTFDNSHNSEEREVGRRTDKRKQI	29
Sme2.5_05438.1_g00004.1	--	-
SlMS10	-------------------------------MEFPSTPFDNSNNSEEREVGRRTDKRKQI	29
Capang02g001895	--	-
Capana02g002096	--	-
CA02g17520	-----------------------MVVIKCGKMEFPKSPFDDSHMSEEREVRGRTDKRKTI	37
AtDYT1	-------------------------------------MGGGSRFQEPVRMSRRKQVTKEK	23
Solyc08g062780.2	HFSENRYMSEMDAYLQKQMMRSSSTQAGIDDESIKHDNGRSNSGSD-SDQNEEEDDPKYR	323
Sotub08g012880.1.1	HFSENRYMSEMDAYLQKQMMRSSSTQAGIDDESVKHDNGRSNSGSD-SDQNEEEDDPKYR	318
Capang00g000803	HFSENRYMSEMDAYLQKQMMRSSSTQSAIDDESVKHDNGRSNSGSD-SDQNEEEDDPKYR	322
Capana08g000254	HFSENRYMSEMDAYLQKQMMRSSSTQSAIDDESVKHDNGRSNSGSD-SDQNEEEDDPKYR	322
CA08g01690	HFSENRYMSEMDAYLQKQMMRSSSTQSAIDDESVKHDNGRSNSGSD-SDQNEEEDDPKYR	322
AtAMS	GMNPFNTVEEDGIPVIGEPSLLVNEQQVVNDKDMNE-NGRVDSGSDCSDQIDDEDDPKYK	304
OsTDR	WPYAGMAVSEPSVAVAQEQMQHAAGGGVAE----SGSEGRKLHGGD----PEDDGDGEGR	275
OsTIP2	AAAATEAYFRGGPRTAGSSSLVFGPADDESAFMVGPFESSPTPRSGGGRKRSRATAG---	168
OsUDT1	-----MPRRARARGGGGGGGEEVKVEDDFIDSVLNFGGGGGGEEDGDDGEEEQQQQQAAA	55

bHLH Domain

AtbHLH089	RKGRGSKKRKIFPTERERRVHFKDRFGDLKNLIPNPTK-NDR-----------ASIVGEA	254
AtbHLH010	RKGRGSRKSRTSPTERERRVHFNDRFFDLKNLIPNPTK-IDR-----------ASIVGEA	274
AtbHLH091	RKGRGKRKNKPFTTERERRCHLNERYEALKLLIPSPSK-GDR-----------ASILQDG	252
Solyc01g081090.2	-KRILGKDIKHHASEKQRRVHFSDKFQALRTLIPNPSK-NNR-----------ATIIADA	234
Sotub01g028050.1.1	-KRELGKEIKHHASEKQRRVHFSDKFQALRTLIPNPSK-NDR-----------ATIIADA	235
Sme2.5_00114.1_g00006.1	-KRELGKDVKHPASEKQRRVHFSDKFQALRTLIPNPSK-NER-----------ASIIADA	239
CA01g26780	-KRE-GNDMKHHASEKLRRIHFSDKFQALRTLIPNPSK-NDR-----------ATIIVDA	244
Capang08g000242	-KRE-GNDMKHHASEKLRRIHFSDKFQALRTLIPNPSK-NDR-----------ATIIADA	244
Solyc01g081100.2	KNRDGIKETKHFATERQRRVHLNDKYKALRSMVPNPSK-NDR-----------ASIVKDA	309
Sotub01g028040.1.1	KNRDGIKETKHFATERQRRVHLNDKYKALRSMVPNPSK-NDR-----------ASIVKDA	308
Sme2.5_00114.1_g00007.1	KNRDGIKETKHFATERQRRVHLNDKFQALRNMVPNPSK-NDR-----------ASIVKDG	203
CA01g26770	KNRDGIKETKHFATERQRRVHLNDKYKALRSMVPNPSK-TDR-----------ASIVKDA	338
Capana01g003305	KNRDGIKETKHFATERQRRVHLNDKYKALRSMVPNPSK-TDR-----------ASIVKDA	316
Capana00g000514	KNRDGIKETKHFATERQRRVHLNDKYKALRSMVPNPSK-TDR-----------ASIVKDA	316
Capang08g000243	KNRDGIKETKHFATERQRRVHLNDKYKALRSMVPNPSK-NDR-----------ASIVKDA	317

```
OsEAT1                     GEFGKGKGKANFATERERREQLNVKFRTLRMLFPNPTK-NDR-----------ASIVGDA   303
Sotub02g022820.1.1         DGEVKEYKSKNLKAERNRRQKLSERLLQLRSLVPNITN-MTK-----------ETIITDA    77
Sme2.5_05438.1_g00004.1    ---------------------------------------MTK-----------ETIITDA    10
SlMS10                     DGEVKEYKSKNLKAERNRRQKLSERLLQLRSLVPNITN-MTK-----------ETIITDA    77
Capang02g001895            ---------------------------------------MTK-----------ETIITDA    10
Capana02g002096            ---------------------------------------MTK-----------ETIITDA    10
CA02g17520                 DGEVKEYKSKNLNAERKRRQKLSERLLELRSLVPNITNAMNQNPFTNFFIQFSKTIITDA    97
AtDYT1                     E-EDENFKSPNLEAERRRREKLHCRLMALRSHVPIVTN-MTK-----------ASIVEDA    70
Solyc08g062780.2           RRNGKGPQSKNLMAERKRRKKLNERLYALRALVPKISK-LDR-----------ASILGDA   371
Sotub08g012880.1.1         RRNGKGPQSKNLMAERKRRKKLNERLYALRALVPKISK-LDR-----------ASILGDA   366
Capang00g000803            RRNGKGPQSKNLMAERKRRKKLNERLYALRALVPKISK-LDR-----------ASILGDA   370
Capana08g000254            RRNGKGPQSKNLMAERKRRKKLNERLYALRALVPKISK-LDR-----------ASILGDA   370
CA08g01690                 RRNGKGPQSKNLMAERKRRKKLNERLYALRALVPKISK-LDR-----------ASILGDA   370
AtAMS                      KKSGKGSQAKNLMAERRRRKKLNDRLYALRSLVPRITK-LDR-----------ASILGDA   352
OsTDR                      SGGAKRQQCKNLEAERKRRKKLNGHLYKLRSLVPNITK-MDR-----------ASILGDA   323
OsTIP2                     -FHGGGPANGVEKKEKQRRLRLTEKYNALMLLIPNRTK-EDR-----------ATVISDA   215
OsUDT1                     AAMGKEFKSKNLEAERRRRGRLNGNIFALRAVVPKITK-MSK-----------EATLSDA   103

AtbHLH089                  IDYIKELLRTIDEFKLLVEKK-----RVKQR-------NREGD-----DVVDE------N   291
AtbHLH010                  IDYIKELLRTIEEFKMLVEKKRCGRFRSKKR-------ARVGEGGGGEDQEEE------E   321
AtbHLH091                  IDYINELRRRVSELKYLVERKRCGG-RHKNNEVDDNNNNKNLDDHGNEDDDDD------D   305
Solyc01g081090.2           IGYIDELKMRVNELKVQVDIKKE---RIKRRRS-------------MVEEYG--------   270
Sotub01g028050.1.1         IGYIHELKMRVNELMIQVDLKKE---RIKRQRS-------------MVEEDD--------   271
Sme2.5_00114.1_g00006.1    IVYINELKMRVNELKIQVDIKKK---RIKRQRT-------------IVEEDG--------   275
CA01g26780                 IGYINELKMRVNDLRNEIDLKKEMM-RMKRQR--------------IVEEDG--------   281
Capang08g000242            IGYINELKMRVNDLKNEIDLKKEMM-RMKRQR--------------IVEEDG--------   281
Solyc01g081100.2           IDYINELKRGVNELKLMAEKKRCNKDRIKRQK----------------TEGGT------T   347
Sotub01g028040.1.1         IDYINELKRGVNELKLMVEKKRCNKDRIKRQK----------------TEGG-------T   345
Sme2.5_00114.1_g00007.1    IDYINELKRGVNELKLMVEKKRCSRDRIKKQK----------------TESG-------T   240
CA01g26770                 IDYINELKRGVNELKLMVEKKRCSKDRIKRQK----------------KEDG--------   374
Capana01g003305            IDYINELKRGVNELKLMVEKKRCSKDRIKRQK----------------KEDG--------   352
Capana00g000514            IDYINELKRGVNELKLMVEKKRCSKDRIKRQK----------------KEDG--------   352
Capang08g000243            IDYINELKRGVNELKLMVEKKRCSKDRIKRQK----------------KEDG--------   353
OsEAT1                     IEYIDELNRTVKELKILVEQKRHGNNRRKVLK-----------------LDQE------A   340
Sotub02g022820.1.1         ITYIRELQMNVDNLSEQLLEMEATHG----------------------------------   103
Sme2.5_05438.1_g00004.1    ITYIRELQMNVDNLSEQLLEMEATHG----------------------------------    36
SlMS10                     ITYIRELQMNVDNLSEQLLEMEATQG----------------------------------   103
Capang02g001895            ITYIRELQTNVDYLSEQLLEMEATHA----------------------------------    36
Capana02g002096            ITYIRELQTNVDYLSEQLLEMEATHA----------------------------------    36
CA02g17520                 ITYIRELQTNVDYLSEQLLEMEATHA----------------------------------   123
AtDYT1                     ITYIGELQNNVKNLLETFHEMEEAPP----------------------------------    96
Solyc08g062780.2           IEYVMELEKQVKDLQLEVEEHSDDDGTGGGRNSDQIHPVVLSHNGTKNRPKSD-NGKLTN   430
Sotub08g012880.1.1         IEYVMELEKQVKDLQLELEEHSDDD--GGGRNPDQIHPVVLSHNGTKNRPKSE-NGKLTN   423
```

Capang00g000803	IEYVMELEKQVKDLQLELEEHSDDE---GRRNQDQIHPDVLSHNGTKNGPKSE-NGKRSN	426
Capana08g000254	IEYVMELEKQVKDLQLELEEHSDDE---GRRNQDQIHPDVLSHNGTKNGPKSE-NGKRSN	426
CA08g01690	IEYVMELEKQVKDLQLELEEHSDDE---GRRNQDQIHPDVLSHNGTKNGPKSE-NGKRSN	426
AtAMS	INYVKELQNEAKELQDELEENSETE----------------DGSNRPQGGMSL-NGTVVT	395
OsTDR	IDYIVGLQKQVKELQDELEDNHVHHK---PPDVLIDHPPPASLVGLDNDDASPPNSHQQQ	380
OsTIP2	IEYIQELGRTVEELTLLVEKKRRREMQGDVVDAATSSVVAG-----MDQAAE-------	263
OsUDT1	IEHIKNLQNEVLELQRQLGDSPG-------------------------------------	126

AtbHLH089	----FKAQSEVVEQCLINKKNNALRCSWLKRKSKFTDVDVRIID-DEVTIKIVQKK----	342
AtbHLH010	DTVNYKPQSEVDQSCFNKNNNNSLRCSWLKRKSKVTEVDVRIID-DEVTIKLVQKK----	376
AtbHLH091	ENMEKKPESDVIDQCS---SNNSLRCSWLQRKSKVTEVDVRIVD-DEVTIKVVQKK----	357
Solyc01g081090.2	AVIMEDNQDDQQVMMNKSTN---WHHQIKSSKNSNTEVDVRIME-DEVIVKFVQHK--QM	324
Sotub01g028050.1.1	AVIMEANQDDQQVMMNKSTN---WHHQIKSSKNTSTEIDVRIME-DEVIVKLVQQK--QI	325
Sme2.5_00114.1_g00006.1	AVIMEAEDD-----INKSIH---CHHQRKSSKNSSTEVDVRIMG-EEVIVKLVQHK--KI	324
CA01g26780	AMIMEADED--QVVMNKSIMSN-WHHQRKCTKNSSTEVDVRVME-DEVSIKLVQQKEMMI	337
Capang08g000242	AMIMEADED--QVVMNKSIMSN-WHHQRKCTKNSSTEVDVRVME-DEVSIKLVQQKEMMI	337
Solyc01g081100.2	ISMDGSDAKQIMDEVEQSYNGNSLRSSWLQRRSKNTEVDVRIVD-DEVTVKLVQQK----	402
Sotub01g028040.1.1	VSMDGTDAKQIMDEVEQSYNGNSLRSSWLQRRSKNTEVDVRIVD-DEVTVKLVQQK----	400
Sme2.5_00114.1_g00007.1	TCVDGTDAKQIMDEVEQSYNGNSLRSSWLQRRSKNTEVDVRIVD-DEVTVKLVQQK----	295
CA01g26770	TSLEGLDAKQ-MDEVEQSYNGNSLRSSWLQRRSKNTEVDVRIVD-DEVTVKLVQQK----	428
Capana01g003305	TSLEGLDAKQ-MDEVEQSYNGNSLRSSWLQRRSKNTEVDVRIVD-DEVTVKLVQQK----	406
Capana00g000514	TSLEGLDAKQ-MDEVEQSYNGNSLRSSWLQRRSKNTEVDVRIVD-DEVTVKLVQQK----	406
Capang08g000243	TSLDGLDAKQ-MDEVEQSYNGNSLRSSWLQRRSKNTEVDVRIVD-DEVTVKLVQQK----	407
OsEAT1	AADGESSSMRPVRDDQDNQLHGAIRSSWVQRRSKECHVDVRIVD-DEVNIKLTEKK----	395
Sotub02g022820.1.1	----EEPETKNEEII-------DTAEEMGKWG-IEPEVQVAHIGPTKLWIKIVCQK----	147
Sme2.5_05438.1_g00004.1	----EELETKNEEII-------DTAEEMGKWG-IEPQVQVAHIGPTKLWIKIVCQK----	80
SlMS10	----EELETKNEEII-------DTADEMGKWG-IEPEVQVANIGPTKLWIKIVCQK----	147
Capang02g001895	----EQLETKNEVII-------DTAEDMGKWG-IEPEVQVAHIGPTKLWIKIVCQK----	80
Capana02g002096	----EQLETKNEVII-------DTAEDMGKWG-IEPEVQVAHIGPTKLWIKIVCQK----	80
CA02g17520	----EQLETKNEVII-------DTAEDMGKWG-IEPEVQVAHIGPTKLWIKIVCQK----	167
AtDYT1	----EIDEEQTDPMIKPEVETSDLNEEMKKLG-IEENVQLCKIGERKFWLKIITEK----	147
Solyc08g062780.2	GS-QREISTNSNGSTDPSRKNQDVEE-NDKLQQMEPQVEVAQLDGNEFFVKVFREH----	484
Sotub08g012880.1.1	GT-HREISTNSNGSADPSRKNQEVEE-NDKLQQMEPQVEVAQLDGNEFFVKVFREH----	477
Capang00g000803	GKGHRELSTNSNGSTDPFRKIQEVEE-NDKLQQMEPQVEVAQLEGNEFFVKVFREH----	481
Capana08g000254	GKGHRELSTNSNGSTDPFRKIQEVEE-NDKLQQMEPQVEVAQLEGNEFFVKVFREH----	481
CA08g01690	GKGHRELSTNSNGSTDPFRKIQEVEE-NDKLQQMEPQVEVAQLEGNEFFVKVFREH----	481
AtAMS	GF-HPGLSCNSN--VPSVKQDVDLENSNDKGQEMEPQVDVAQLDGREFFVKVICEY----	448
OsTDR	PPLAVSGSSSRRSNKDPAMTDDKVGGGGGGGHRMEPQLEVRQVQGNELFVQVLWEH----	436
OsTIP2	SSEGEVMAAAAMGAVAPPPRQAPIRSTYIQRRSKETFVDVRIVE-DDVNIKLTKRR----	318
OsUDT1	----EAWEKQCSASCSESFVP-------TENAHYQGQVELISLGSCKYNLKIFWTK----	171

AtbHLH089	--KINCLLFVSKVVDQLELDLHHVAGAQIGEHHSFLFNAKISEGSSVYAS---AIADRVM	397

```
AtbHLH010                 --KINCLLFTTKVLDQLQLDLHHVAGGQIGEHYSFLFNTKICEGSCVYAS---GIADTLM   431
AtbHLH091                 --KINCLLLVSKVLDQLQLDLHHVAGGQIGEHYSFLFNTKIYEGSTIYAS---AIANRVI   412
Solyc01g081090.2          LKGVNCLLLVSKALDELQLDLQHVAGGLIGDHYSYLLNSKICEGCTVYAS---VIANKVI   381
Sotub01g028050.1.1        LKGVNCLLLVSKTLDELQLDLKHVAGGLIGDHYSYLLNSKICEGCTVYAS---VVANKVI   382
Sme2.5_00114.1_g00006.1   IKKMNCLVFVSKAFDDLQLDLQHVAGGLIGDQYSYLFNSKICEGCTVYAS---EVANKVI   381
CA01g26780                KKRMNCLVFVSKALDELRLDLQHVAGGLIGDHYSYLFNSKICEGCTVYAS---AIANKVI   394
Capang08g000242           KKRMNCLVFVSKALDELRLDLQHVAGGLIGDHYSYLFNSKV-------------------   378
Solyc01g081100.2          --RINCLFSASKVLDDLQLDLHHVAGGLIGDYYSFLFNSKISEGSTVYAS---AIAKKLI   457
Sotub01g028040.1.1        --RINCLFFASKVLDDLQLDLHHVAGGLIGDYYSFLFNSKISEGSTVYAS---AIAKKLI   455
Sme2.5_00114.1_g00007.1   --RINCLFFASKVLDELQLDLHHVAGGLIGDYYSFLFNSKISEGSTVYAS---AIAKKLI   350
CA01g26770                --RINCLLFASKVLDELQLDLHHVAGGLIGDYYSFLFNSKISEGSTVYAS---AIAKKLI   483
Capana01g003305           --RINCLLFASKVLDELQLDLHHVAGGLIGDYYSFLFNSKISEGSTVYAS---AIAKKLI   461
Capana00g000514           --RINCLLFASKVLDELQLDLHHVAGGLIGDYYSFLFNSKISEGSTVYAS---AIAKKLI   461
Capang08g000243           --RINCLLFASKVLDELQLDLHHVAGGLIGDYYSFLFNSKISEGSTVYAS---AIAKKLI   462
OsEAT1                    --KANSLLHAAKVLDEFQLELIHVVGGIIGDHHIFMFNTKVSEGSAVYAC---AVAKKLL   450
Sotub02g022820.1.1        --KRGGLTKLMEAMNVLGFDINDTSATASKGAILITTSVEVVRGGLTEAN---RIREILL   202
Sme2.5_05438.1_g00004.1   --KRGGFTKLMEAMNVLGFDINDTSVTASKGALLVTSSVEVVRGGLTEAD---RIREILL   135
SlMS10                    --KRGGLTKLMEAMNALGFDINDTSATASKGAILITSSVEVVRGGLTEAN---RIREILL   202
Capang02g001895           --KRGGFTKLMETMNVLGFDLNDTSVTASRGALLVTASVEVVRGGLNEAN---QIREILL   135
Capana02g002096           --KRGGFTKLMETMNVLGFDLNDTSVTASRGALLVTASVEVVRGGLNEAN---QIREILL   135
CA02g17520                --KRGGFTKLMETMNVLGFDLNDTSVTASRGALLVTASVEVGETYVKKMNFLVAMKNYCL   225
AtDYT1                    --RDGIFTKFMEVMRFLGFEIIDISLTTSNGAILISASVQTQELCDVEQT-----KDFLL   200
Solyc08g062780.2          --KAGGFVRTLEALNSLGLEVTNVNATRHTCLVSSIFKVEQKRDNEMVQADH--VRDTLL   540
Sotub08g012880.1.1        --KAGGFVRTLEALNSLGLEVTNVNATRHTCLVSSIFKVEQKRDNEMVQADH--VRDTLL   533
Capang00g000803           --KTGGFVRILEALNSLGLEVTNVNATRHTCLVSNIFKVE-KRDNEIVQADH--VRDTLL   536
Capana08g000254           --KTGGFVRILEALNSLGLEVTNVNATRHTCLVSNIFKVE-KRDNEIVQADH--VRDTLL   536
CA08g01690                --KTGGFVRILEALNSLGLEVTNVNATRHTCLVSNIFKVE-VSD---IFFNH--SHPTPY   533
AtAMS                     --KPGGFTRLMEALDSLGLEVTNANTTRYLSLVSNVFKVE-KNDNEMVQAEH--VRNSLL   503
OsTDR                     --KPGGFVRLMDAMNALGLEVINVNVTTYKTLVLNVFRVMVRDSEVAVQADR--VRDSLL   492
OsTIP2                    --RDGCLAAASRALDDLRLDLVHLSGGKIGDCHIYMFNTKIHSGSPVFAS---AVASRLI   373
OsUDT1                    --RAGLFTKVLEALCSYKVQVLSLNTISFYGYAESFFTIEVKGEQDVVMVELRSLLSSIV   229

AtbHLH089                 EVLKKQYMEALSANNGYHCYSSD-------------------------------------   420
AtbHLH010                 EVVEKQYMEAVPSN-GY-------------------------------------------   447
AtbHLH091                 EVVDKHYMASLPNS-NY-------------------------------------------   428
Solyc01g081090.2          DVLDKEHADIN-------------------------------------------------   392
Sotub01g028050.1.1        EVLDKKHAEIN-------------------------------------------------   393
Sme2.5_00114.1_g00006.1   EVLDKEHEDIN-------------------------------------------------   392
CA01g26780                EVLDKEHATIN-------------------------------------------------   405
Capang08g000242           ------------------------------------------------------------     -
Solyc01g081100.2          EVVDIQYAAIAPTNSY--------------------------------------------   473
Sotub01g028040.1.1        EVVDIQYAAIAPTNSY--------------------------------------------   471
Sme2.5_00114.1_g00007.1   EVVDIQYAAIAPTNSY--------------------------------------------   366
```

```
CA01g26770                 EVVDIQYAAIAPANSY-------------------------------------------   499
Capana01g003305            EVVDIQYAAIAPANSY-------------------------------------------   477
Capana00g000514            EVVDIQYAAIAPANSY-------------------------------------------   477
Capang08g000243            EVVDIQYAAIAPANSY-------------------------------------------   478
OsEAT1                     QAVDVQHQALDIFN---------------------------------------------   464
Sotub02g022820.1.1         EIIHGIY----------------------------------------------------   209
Sme2.5_05438.1_g00004.1    EIIHGIY----------------------------------------------------   142
SlMS10                     EIIHGIY----------------------------------------------------   209
Capang02g001895            EIIRGI-----------------------------------------------------   141
Capana02g002096            EIIRGI-----------------------------------------------------   141
CA02g17520                 NMSGFVVVEKRYLA---------------------------------------------   239
AtDYT1                     EVMRSNP----------------------------------------------------   207
Solyc08g062780.2           ELTRNPSRGWSEMGRASSDNINNNNANGTTDYHQHQLHDHHLDNNNQHKQTNSHHFHTHH   600
Sotub08g012880.1.1         ELTRNPSRGWSEMGRASSDNNNNNNANGTTDYHQHQHHDHHLDNNNQHKQTNSHHFHRHH   593
Capang00g000803            ELTRNPSRGWSEMARASSDNNANGCTE--YLHNQHHHHDHHLDN-NHHKQANSHRYQRHH   593
Capana08g000254            ELTRNPSRGWSEMARASSDNNANGCTE--YLHNQHHHHDHHLDN-NHHKQANSHRYQRHH   593
CA08g01690                 STT-------------------------------HKEKKNLRP-TFTKRIA--------   552
AtAMS                      EITRNTSRGWQDDQMATGSMQNEKNEVDYQHYDDHQHHNGHHHPFDHQMNQSAHHHHHHQ   563
OsTDR                      EVTRETYPGVWPSPQEEDDAKFDGGDG------GQAAAAAAAAGGEHYHDEVGGGYHQHL   546
OsTIP2                     EVVDEY-----------------------------------------------------   379
OsUDT1                     EVPSI------------------------------------------------------   234


AtbHLH089                  ---------     -
AtbHLH010                  ---------     -
AtbHLH091                  ---------     -
Solyc01g081090.2           ---------     -
Sotub01g028050.1.1         ---------     -
Sme2.5_00114.1_g00006.1    ---------     -
CA01g26780                 ---------     -
Capang08g000242            ---------     -
Solyc01g081100.2           ---------     -
Sotub01g028040.1.1         ---------     -
Sme2.5_00114.1_g00007.1    ---------     -
CA01g26770                 ---------     -
Capana01g003305            ---------     -
Capana00g000514            ---------     -
Capang08g000243            ---------     -
OsEAT1                     ---------     -
Sotub02g022820.1.1         ---------     -
Sme2.5_05438.1_g00004.1    ---------     -
SlMS10                     ---------     -
Capang02g001895            ---------     -
Capana02g002096            ---------     -
```

CA02g17520	--------	-
AtDYT1	--------	-
Solyc08g062780.2	HH------	602
Sotub08g012880.1.1	HH------	595
Capang00g000803	HH------	595
Capana08g000254	HH------	595
CA08g01690	--------	-
AtAMS	HINHYHNQ	571
OsTDR	HYLAFD--	552
OsTIP2	--------	-
OsUDT1	--------	-

Figure S2 Multiple sequence alignment of bHLH proteins related to pollen and tapetum development based on their full-length protein sequences. Entire protein sequences were aligned using Clustal X ver. 2 (Larkin *et al.*, 2007) with default settings, and the conserved amino acids were shaded using GeneDoc (2.6) (Nicholas *et al.*, 1997). The bHLH domain was marked above the alignment.

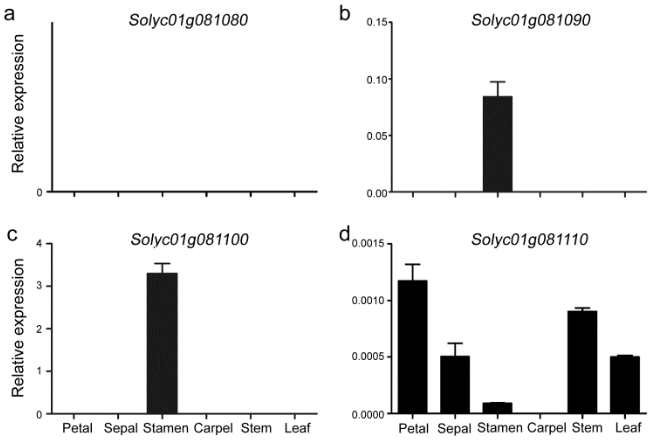

Figure S3 The relative expression patterns of the genes within the fine-mapped region of the *ms32* locus in the WT.

Each value represents the mean ± SE of three replicates.

```
OsEAT1                   -----MIVGAGYFEDSHDQSLMAGSLIHDSNQAPASSENTSIDLQKFKVHPYSTEALSNTANLAE     60
AtbHLH089                MGGGGMFEEIGCFDPNAPAEMTAESSFSPSEPPP---TITVIGSNSNS-NCS--LEDLSAFHLSP     59
AtbHLH010                ---------MGCFDPNTPAEVTVESSFSQAEPPPPPPQVLVAGSTSNS-NCSVEVEELSEFHLSP     55
AtbHLH091                -----MYEESSCFDPNSMVDNNGGFCAAETTFTVSHQFQPPLGSTTNSFDDDLKLPTMDEFSVFP     60
Solyc01g081100.2         ----MYVEESVCYDPATHHVQHEGLTEDVFVIQEHTYHNNNDSSQQDVAVAAAAAALEIEFQHQL     61
Solyc01g081100.2ms       ----MYVEESVCYDPATHHVQHEGLTEDVFVIQEHTYHNNNDSSQQDVAVAAAAAALEIEFQHQL     61
Solyc01g081090.2         ----MYASQSLSFHPTSDQEDEKSFVQNHIAASGFVELQQQ---QQQFHSSENNLNMQLEFGHDS     58
OsTIP2                   -----------MYHPQCELLMPLESLEMDVGQSHLAAAVAAAMPGELNFHLLHSLDAAAAAASST     54

OsEAT1                   AARAINHLQHQLEIDLEQEVPPVETANWDPAICTIPDHIINHQFSEDPQNILVEQQIQQYDSALY    125
AtbHLH089                QDSSLPASASAYAHQLHINATPN--------CDHQFQSSMHQTLQDPSYAQQSNHWDNGYQDFVN    116
AtbHLH010                QDC---PQASSTPLQFHINPPPPPPP----PCDQLHNNLIHQMA---SHQQQHSNWDNGYQDFVN    110
AtbHLH091                SVIS---LPNSETQNQNIS---------------NNNHLINQMIQE-SNWGVSEDNSNFFMNTSH    106
Solyc01g081100.2         N-----------------------------------------------LEMEQCYNNNNNNNTH     78
Solyc01g081100.2ms       N-----------------------------------------------LEMEQCYNNNNNNNTH     78
Solyc01g081090.2         NNTR-------------------------------------------FHPNWEEISFNPYNNQQL     80
OsTIP2                   AASAS-------------------------------------------SQPTVDYFFGGADQQP     75

OsEAT1                   PNGVYTPAPDLLNLMQCTMAPAFPATTSVFGDTTLNGTNYLDLN---------------------    169
AtbHLH089                LGPN-HTTPDLLSLLQLPRSSLPPFANP-------------------------------------    143
AtbHLH010                LGPNSATTPDLLSLLHLPRCSLPPNHHPSS-----------------------------------    140
AtbHLH091                PNTTTTPIPDLLSLLHLPRCSMS------------------------------------------    129
Solyc01g081100.2         NNNNNIVNEGLSCDQANWGEMNFPPYQNQQHNDNGNSNNNFHQQDFSNPISETPYLTTPDLLNMF    143
Solyc01g081100.2ms       NNNNNIVNEGLSCDQAN------------------------------------------------     95
Solyc01g081090.2         SYPISNPSLGLLGGFHQR-----------------------------------------------     98
OsTIP2                   PPPAAMQYDQLAAPHHHQTVAMLR-----------------------------------------     99

OsEAT1                   ----------------------GELTGVAAVPDSGSGLMFASDSALQLGYHGTQSHLIKDICHSL    212
AtbHLH089                --------------------------SIQDIIMTTSSSVAAYDPLFHLNFP-LQPP------NGS    175
AtbHLH010                ----------------------MLPTSFSDIMSSSSAAAVMYDPLFHLNFP-MQPRDQNQLRNGS    182
AtbHLH091                -------------------------LPSSDIMAG---SCFTYDPLFHLNLP-PQPPLIPSNDYSG    165
Solyc01g081100.2         PLPRCTQSSLLPQKSPNLLTSLGLIGDIDGGGASTSSAICDPSSLLLPLNLPPQPPLLRELFHSF    208
Solyc01g081100.2ms       -----------------------------------------------------------------      -
Solyc01g081090.2         ---------------------------TELASTSTNLFYEPPQMNMPLNLCTPQSSLFKELFHLS    136
OsTIP2                   -------------------------DYYGGHYPPAAAAAAATEAYFRGGPR-------------T    126

OsEAT1                   PQNYGLFPSEDERDVIIGVGSGDLFQEIDDRQFDSVLECRRGKGEF---------GKGKGKAN-F    267
AtbHLH089                FMG--VDQDQTETNQGVNLMYDE--ENNN---------LDDGLNRK---------GRGSKKRKIF    218
AtbHLH010                CLLGVEDQIQMDANGGMNVLYFEGANNNNGGFENEILEFNNGVTRK---------GRGSRKSRTS    238
AtbHLH091                YLLGIDTNTTTQRDE--SNVGDE---NNNAQFDSGIIEFSKEIRRK---------GRGKRKNKPF    216
Solyc01g081100.2         PHGYGLRNLRSNNNTSFFNGLEETDQGLYQENGETRPFQNGIFEFSGGMNDIAKNRDGIKETKHF    273
Solyc01g081100.2ms       -----------------------------------------------------------------      -
```

```
Solyc01g081090.2      PHGSSYGLGSSGTGSLFSLGHDQEEVTGNLYHDGSFHELTGDMMIN---SAAIKKRILGKDIKHH   198
OsTIP2                AGSSSLVFGPADDESAFMVGPFESSPTPRSGGGRKRSRATAGFHGG------------GPANGVE   179

                                 bHLH domain

OsEAT1                ATERERREQLNVKFRTLRMLFPNPTKNDRASIVGDAIEYIDELNRTVKELKILVEQKRHGNNRRK   332
AtbHLH089             PTERERRVHFKDRFGDLKNLIPNPTKNDRASIVGEAIDYIKELLRTIDEFKLLVEKK-----RVK   278
AtbHLH010             PTERERRVHFNDRFFDLKNLIPNPTKIDRASIVGEAIDYIKELLRTIEEFKMLVEKKRCGRFRSK   303
AtbHLH091             TTERERRCHLNERYEALKLLIPSPSKGDRASILQDGIDYINELRRRVSELKYLVERKRCGG-RHK   280
Solyc01g081100.2      ATERQRRVHLNDKYKALRSMVPNPSKNDRASIVKDAIDYINELKRGVNELKLMAEKKRCNKDRIK   338
Solyc01g081100.2ms    -----------------------------------------------------------------     -
Solyc01g081090.2      ASEKQRRVHFSDKFQALRTLIPNPSKNNRATIIADAIGYIDELKMRVNELKVQVDIKKERIKRRR   263
OsTIP2                KKEKQRRLRLTEKYNALMLLIPNRTKEDRATVISDAIEYIQELGRTVEELTLLVEKKRRRREMQG   244

OsEAT1                VL-----------------KLDQEAAADGESSSMRPVRDDQDNQLHGAIRSSWVQRRSKECHVDV   380
AtbHLH089             QR-------NREGD-----DVVDEN----FKAQSEVVEQCLINKKNNALRCSWLKRKSKFTDVDV   327
AtbHLH010             KR-------ARVGEGGGGEDQEEEEDTVNYKPQSEVDQSCFNKNNNNSLRCSWLKRKSKVTEVDV   361
AtbHLH091             NNEVDDNNNNKNLDDHGNEDDDDDDENMEKKPESDVIDQCS---SNNSLRCSWLQRKSKVTEVDV   342
Solyc01g081100.2      RQ----------------KTEGGTTISMDGSDAKQIMDEVEQSYNGNSLRSSWLQRRSKNTEVDV   387
Solyc01g081100.2ms    -----------------------------------------------------------------     -
Solyc01g081090.2      SM----------------VEEYGAVIMEDNQDDQQVMMNKSTNWHHQIKSS-----KNSNTEVDV   307
OsTIP2                DVVD------AATSSVVAGMDQAAESSEGEVMAAAAMGAVAPPPRQAPIRSTYIQRRSKETFVDV   303

OsEAT1                RIVDDEVNIKLTEK----KKANSLLHAAKVLDEFQLELIHVVGGIIGDHHIFMFNTKVSEGSAVY   441
AtbHLH089             RIIDDEVTIKIVQK----KKINCLLFVSKVVDQLELDLHHVAGAQIGEHHSFLFNAKISEGSSVY   388
AtbHLH010             RIIDDEVTIKLVQK----KKINCLLFTTKVLDQLQLDLHHVAGGQIGEHYSFLFNTKICEGSCVY   422
AtbHLH091             RIVDDEVTIKVVQK----KKINCLLLVSKVLDQLQLDLHHVAGGQIGEHYSFLFNTKIYEGSTIY   403
Solyc01g081100.2      RIVDDEVTVKLVQQKRIN----CLFSASKVLDDLQLDLHHVAGGLIGDYYSFLFNSKISEGSTVY   448
Solyc01g081100.2ms    -----------------------------------------------------------------     -
Solyc01g081090.2      RIMEDEVIVKFVQHKQMLKGVNCLLLVSKALDELQLDLQHVAGGLIGDHYSYLLNSKICEGCTVY   372
OsTIP2                RIVEDDVNIKLTKR----RRDGCLAAASRALDDLRLDLVHLSGGKIGDCHIYMFNTKIHSGSPVF   364

OsEAT1                ACAVAKKLLQAVDVQHQALDIFN---------   464
AtbHLH089             ASAIADRVMEVLKKQYMEALSANNGYHCYSSD   420
AtbHLH010             ASGIADTLMEVVEKQYMEAVPSN-GY------   447
AtbHLH091             ASAIANRVIEVVDKHYMASLPNS-NY------   428
Solyc01g081100.2      ASAIAKKLIEVVDIQYAAIAPTNSY-------   473
Solyc01g081100.2ms    --------------------------------     -
Solyc01g081090.2      ASVIANKVIDVLDKEHADIN------------   392
OsTIP2                ASAVASRLIEVVDEY-----------------   379
```

Figure S4 Multiple sequence alignment of Solyc01g081100.2 and its homologs in Arabidopsis and rice based on their full-length protein sequences. Entire protein sequences were aligned using Clustal X ver. 2 (Larkin *et al.*, 2007) with

default settings, and the conserved amino acids were shaded using GeneDoc (2.6) (Nicholas *et al.*, 1997). The bHLH domain was marked above the alignment. Solyc01g081100.2ms presents the predicted Solyc01g081100.2 protein in *ms32* mutant.

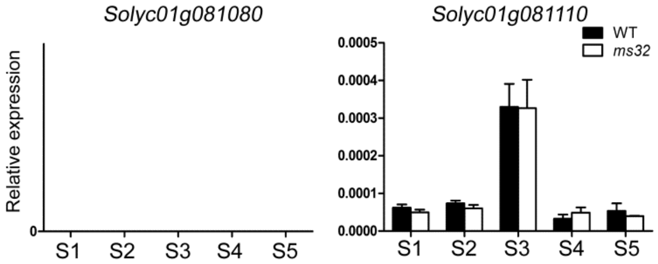

Figure S5 Transcription expression of *Solyc01g081080* and *Solyc01g081110* in flower buds of WT and *ms32* plants. Each value represents the mean ± SE of three replicates.

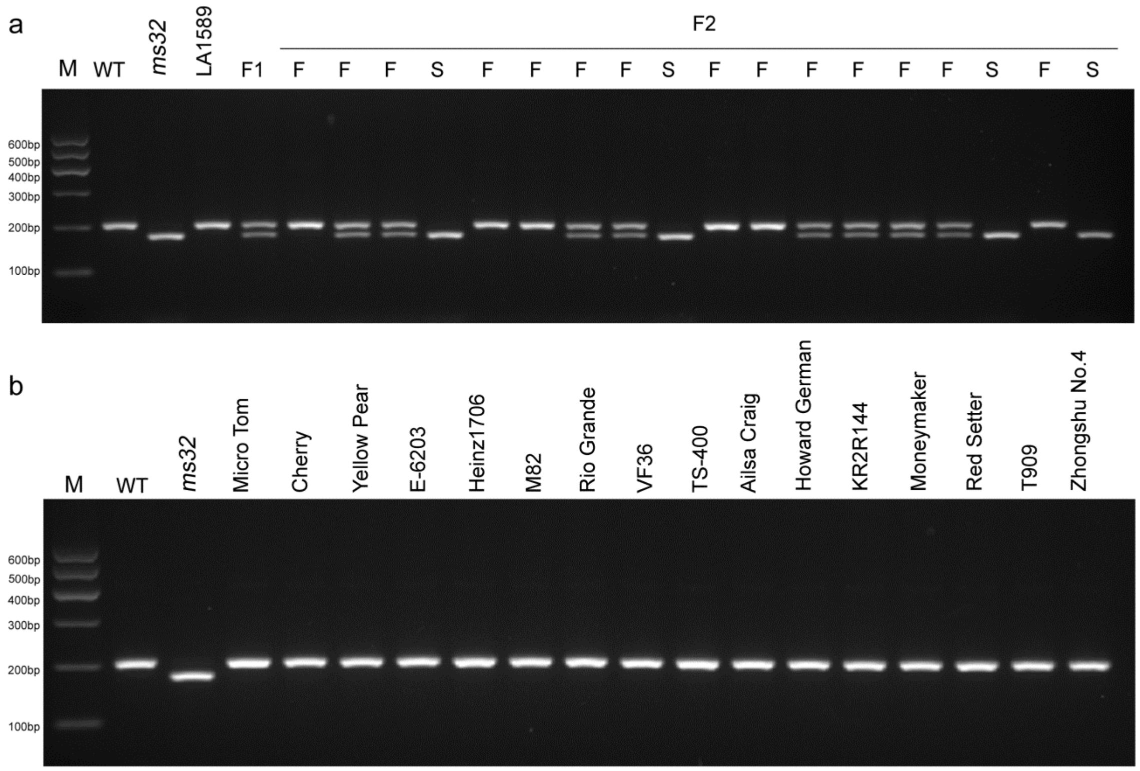

Figure S6 Agarose gel electrophoresis of PCR products after digestion with DdeI using the *MS32* candidate gene-specific marker MS32D. a Agarose gel electrophoresis of the DdeI digested PCR fragments amplified from LA3132, LA1589, F_1, and F_2 plants using the marker MS32D. M, DNA marker; F, male fertile; S, male sterile. b Agarose gel electrophoresis of the DdeI digested PCR fragments amplified from 18 tomato lines using the marker MS32D. They are male fertile lines except *ms32*.

Table S1 Primers used in this study

Primer name	Purpose	Forward sequence (5'→3')	Reverse sequence (5'→3')	SGN ID	Comments
HP107	Fine mapping of *ms32*	GTTGGTCCTGCAGCTAAGAG	AACCTTAACCCTCCAGCAAC		InDel marker
HP109	Fine mapping of *ms32*	GTATCTTTCTCCTAAGATGATG	CCCTTCGATAAGGTTTTTCAC		InDel marker
HP119	Fine mapping of *ms32*	CCATGATCATCAAGAACTAC	CTAGAACAGTAGAAGGGATAC		InDel marker
HP4547	Fine mapping of *ms32*	CTCCACGTCATCACTTTGCC	GTTGGTATGCTCTAAGTTATGGAAA		InDel marker
HP1693	Fine mapping of *ms32*	CAAGTACCAAACTTTTTAGCCAC	CAACATTGAATGAAACGGGTG		InDel marker
HP129	Fine mapping of *ms32*	CCCTCACTGAATGGACAATG	GAACTACTACTTCTTATGCACG		InDel marker
HP4555	Fine mapping of *ms32*	TCGTGCTAAAAGGCTCATAGGTA	CCAATGAGAGCTGAGAGTCTGT		InDel marker
LXY1	Fine mapping of *ms32*	GGGGGTAATTAAGACTACATA	TTCAAGTTTGCCCATCATTTT		InDel marker
MS32D	Fine mapping of *ms32*	GATGATTGCGTGCATCTTGG	CGAAGGGTTATCTTGTGATCAAGCGACTT		dCAPS marker, digested by DdeI
LXY5	Fine mapping of *ms32*	CTCGGTGTTTGATAGGATTGGGTAT	TTTTCCGCATCCGCTTCTCCGCATT		InDel marker
xy1	Sequence of the candidate gene	AACTAGCTGATCACCTGAATACATA	TCTCAATTGCTGACTACTTGTTTGT	Solyc01g081100	
xy3	Sequence of the candidate gene	ACTATCATCCACTCTGGCTGC	GACCATCAAATAACTCTTGGCAT	Solyc01g081100	
xy5	Sequence of the candidate gene	AGTTAAGGACAGTTTTGTCAGTCT	TCATGGCTTCAGAGAAGGTCT	Solyc01g081100	
xy7	Sequence of the candidate gene	TCACCAATAAGTCCACCAGCAA	TACCAAGATGCACGCAATCA	Solyc01g081100	
xy9	Sequence of the candidate gene	TAGCCATGTGGAAAAGAGTGGA	TCAGTCTCCTAAACCAAAACCAACC	Solyc01g081100	
xy11	Sequence of the candidate gene	ACTTCTGCAGACCTGTGAAACA	TGACAATGGGGTGGGACAAG	Solyc01g081100	
xy13	Sequence of the candidate gene	CTCGGTGTTTGATAGGATTGGG	ACGGGGAGCGTGTTATCTTT	Solyc01g081100	
xy15	Sequence of the candidate gene	TACGTGTGAAAGATGTAAGTATGGC	GACGTTGGCAGGGGAAATC	Solyc01g081100	
mp1	Sequence of the candidate gene	TCACGACTCCAAATCTCATTCCA	GCCTGAAATCTCATTGATACACT	Solyc01g081090	
mp3	Sequence of the candidate gene	CAATTATCTTTCGGGACAAATAC	TCCAATCAACAATCTTAAATGGCA	Solyc01g081090	
mp5	Sequence of the candidate gene	CACTAAAATGTATTTTCGCTGAA	CTATTTACATTAATCCTTCTCCTTC	Solyc01g081090	
mp7	Sequence of the candidate gene	CGATCCATATCCCTTTTTGA	AGAGCAGAGAGTCTGTCGAA	Solyc01g081090	
mp9	Sequence of the candidate gene	TGTAGATTCATTGCATAGCAAAACT	CATCTGGATTCGTGGAGCTG	Solyc01g081090	
mp11	Sequence of the candidate gene	CTTCCCAATTTGGATGAAACCTT	GTAACCACGTTGACTAACAGGCAA	Solyc01g081090	
mp13	Sequence of the candidate gene	ACCATCGTTTTCTGTGTCCAGT	GTCTACAATTGTTATCATTAGGG	Solyc01g081090	
mp15	Sequence of the candidate gene	AACGGTATATTGTTTCTATCC	CAACAAGTCGTACAAGTATG	Solyc01g081090	
LJ1	Sequence of the candidate gene	TCCATAACATAAAGAATGCCCAAAAG	GGAAATCAGAACCCCATCATC	Solyc01g081080	
LJ3	Sequence of the candidate gene	TATTAACCAAGCAAAGTGATGGGG	GTGTGCTCCTCTACTCTCATCTGC	Solyc01g081080	

LJ5	Sequence of the candidate gene	GTGTCAAGAAACTATAACCATAGGTG	AAATCAGCACAATTGCCAAGAATC	Solyc01g081080	
LJ7	Sequence of the candidate gene	TGCGTTCTCGAAGCCTTTATC	AGTTAATAGTTCATTTTTTAGGAGTTTG	Solyc01g081080	
LJ9	Sequence of the candidate gene	GGATAATAAACTAGCAGCAAACATAATAT	CTTGGAGTAAAATTAAACCTTCTGTAA	Solyc01g081080	
LJ11	Sequence of the candidate gene	CATCACTATAACCAAGAAAAACACATTT	GGCACAAAGGCGAGCCC	Solyc01g081080	
LJ13	Sequence of the candidate gene	ACATACCATTCATTGCTTTTTGTTA	CTAAGCGTAGACATCATAACTATTCGTC	Solyc01g081080	
LJ15	Sequence of the candidate gene	TTTACTTGATCCATGTACTACGAAAT	TTGAAATTCATAGCACAACAGGAA	Solyc01g081110	
LJ17	Sequence of the candidate gene	ACGTTACTTGAAGTGCCAGGTAGC	GATACCACAAATGTGAAAGATACGACA	Solyc01g081110	
LJ19	Sequence of the candidate gene	TTCCGAAAAAAAGTTGTTGAAGGATA	CAGCTGTTTCTGCTTGTTGTCTACG	Solyc01g081110	
LJ21	Sequence of the candidate gene	GAATTTTCACTAAGAAATAAACGCA	CAACTTGGATTGATCGCAGTAA	Solyc01g081110	
RTP	RT-PCR for candidate gene	CTGCAGAAGTAGAACAGAAGAAC	CCATAAATGAAAGAGCTGAAAAC	Solyc01g081100	
Solyc01g081080	qPCR for gene expression analysis	ATTGACCATTGGTAAGGTAACATTCTTTTT	CCCATCATCAGCAAAGGGGATAGAT	Solyc01g081080	Developed in this study
Solyc01g081090	qPCR for gene expression analysis	GGGACGGGTTCTTTGTTTAGCCTTG	CTTTACCTAATATCCTCTTCTTAATAGC	Solyc01g081090	Developed in this study
Solyc01g081100	qPCR for gene expression analysis	TGTTTCCATTACCAAGATGC	GGGGGTTGTGGGGGTAGATT	Solyc01g081100	Developed in this study
Solyc01g081110	qPCR for gene expression analysis	ACCATCATCCTCTCGTCAGCCCTTA	TTTGCGTTGGATTTGTATGTAGTGAACA	Solyc01g081110	Developed in this study
SlMS10	qPCR for gene expression analysis	AGATCTCTCTGATTCGATTAGCTTCAG	TCTTGAAATGGAAGCAACTCAGG	Solyc02g079810	Jeong *et al.*,2014
AtAMS-like	qPCR for gene expression analysis	TGCAGAGATGTTATGTTTCAGCATC	TCGTCTCTGTCTCTTTCTCCTTCTG	Solyc08g062780	Jeong *et al.*,2014
AtMYB103-like	qPCR for gene expression analysis	ACAAATTACCTTAGGCCTGATCTCAAACA	AATTCCCATACCAGATAATTTCTTTTTGAG	Solyc10g005760	Developed in this study
AtMS1-like-1	qPCR for gene expression analysis	GGGCGTCTTTGCTACAATCCCAAC	ATCCATCCTTGATTGCCAACATAATCG	Solyc04g008420	Jeong *et al.*,2014
AtTDF1-like1	qPCR for gene expression analysis	GAACGGATAATGATGTGAAGAACCT	CTGGTCTAGACATAAATGCACCTTTT	Solyc03g113530	Developed in this study
AtTDF1-like2	qPCR for gene expression analysis	GACCAATTATCTGCGTCCCGAT	GTCTGTTCTCCCTGGTAATTGTTTCG	Solyc03g059200	Developed in this study
SlGAS100	qPCR for gene expression analysis	TATATAGACATGGCAATGAAATGGC	AGTCAAGACAACGATCAAGAATGC	Solyc06g064470	Perez-Martin *et al.*, 2018
TA29	qPCR for gene expression analysis	AAGATTTTAACCATGAACTTCTTC	ACATTCTTCAGTGTCACATACATC	Solyc02g078370	Jeong *et al.*,2014
Cysteine protease	qPCR for gene expression analysis	ATTGGTGTCGATTGGAGGAAG	CAAATGCACTTTCCATAAACCC	Solyc07g053460	Jeong *et al.*,2014
Asparatic protease-1	qPCR for gene expression analysis	GTGATATTAATTGGCTTCAATGTGAACC	ATACTCGCCGGAACCTGTAACATC	Solyc06g069220	Jeong *et al.*,2014
Asparatic protease-2	qPCR for gene expression analysis	AAACAAGCGGGTGCAACGGTCA	TTTCAAAATTTCGCCACAACCAAA	Solyc08g068870	Developed in this study
Arabinogalactan protein	qPCR for gene expression analysis	CCAAGAACTATAAGATCACCCTTTTCATTC	CCGAAAAAATACGTCACTAACAACCTTTG	Solyc11g072780	Developed in this study
Sister chromatid cohesion	qPCR for gene expression analysis	AGTGAGATCATGAGAATTACAGCTCC	GATGAAGTTTGACAGCACTTTCTTG	Solyc03g116930	Jeong *et al.*,2014
TomA108	qPCR for gene expression analysis	ATGCAATTAGGAGCCTTGATTC	CAGTTCCAGTTCCTGTTCCG	Solyc01g009590	Perez-Martin *et al.*, 2018
Endo-1,3-β-glucanase	qPCR for gene expression analysis	AAAAAGATTACTACGCGAGTCAAAACATTT	GACGGATCAGGAAGGACAGTAGATTTT	Solyc03g046200	Developed in this study
LeGPR92	qPCR for gene expression analysis	ATGCAATTAGGAGCCTTGATTC	CAGTTCCAGTTCCTGTTCCG	Solyc02g032910	Jeong *et al.*,2014
LAT52	qPCR for gene expression analysis	AAGGTGTGACTGATAAAGATGGC	AACCCAACTCATCAAGAGCTTC	Solyc10g007270	Jeong *et al.*,2014

Lipid transfer protein-1	qPCR for gene expression analysis	AACAATTCGCAGCGTCTGATAAGCC	CCTTCAAACCGTGGTGCAATCAAC	Solyc06g059790	Developed in this study
Lipid transfer protein-2	qPCR for gene expression analysis	GCTGATCTATGCTATGGTCATTCTGTTGT	CAGAATAATCGCCGCGCTTTTTC	Solyc01g095780	Developed in this study
AtMS2 -like	qPCR for gene expression analysis	ACTGGAGGACCAACCCGCCTTAT	CTCCAATGCAGAATGGCTTCTCC	Solyc03g051960	Developed in this study
SlACTIN	qPCR for gene expression analysis	GGGATGGAGAAGTTTGGTGGTGG	CTTCGACCAAGGGATGGTGTAGC	Solyc03g078400	Qiu *et al.*,2016

References

Jeong HJ, Kang JH, Zhao MA, Kwon JK, Choi HS, Bae JH, Lee HA, Joung YH, Choi D, Kang BC (2014) Tomato *Male sterile 10*^{35} is essential for pollen development and meiosis in anthers. J Exp Bot 65(22):6693-6709.

Larkin MA, Blackshields G, Brown NP, Chenna R, McGettigan PA, McWilliam H, Valentin F, Wallace IM, Wilm A, Lopez R, Thompson JD, Gibson TJ, Higgins DG (2007) Clustal W and Clustal X version 2.0. Bioinformatics 23, 2947-2948.

Nicholas KB, Nicholas HBJ, Deerfield DWI (1997) GeneDoc: analysis and visualization of genetic variation. EMBNEW NEWS 4, 14.

Qiu Z, Wang X, Gao J, Guo Y, Huang Z, Du Y (2016) The Tomato *Hoffman's Anthocyaninless* Gene Encodes a bHLH Transcription Factor Involved in Anthocyanin Biosynthesis That Is Developmentally Regulated and Induced by Low Temperatures. PloS ONE 11, e0151067.
